# Tick hazard in the South Downs National Park (UK): species, distribution, key locations for future interventions, site density, habitats

**DOI:** 10.7717/peerj.17483

**Published:** 2024-06-12

**Authors:** Jo Middleton, Ian Cooper, Anja S. Rott

**Affiliations:** 1 Ecology and Evolution, School of Life Sciences, University of Sussex, Falmer, United Kingdom; 2 Department of Primary Care and Public Health, Brighton and Sussex Medical School, University of Sussex, Falmer, United Kingdom; 3 Centre for Precision Health and Translational Medicine; Centre for Regenerative Medicine and Devices, School of Applied Sciences, University of Brighton, Brighton, United Kingdom; 4 Ecology, Conservation and Society Research and Enterprise Group, School of Applied Sciences, University of Brighton, Brighton, United Kingdom

**Keywords:** Ticks, Acarology, National parks, Tick hazard, *Ixodes ricinus*, *Haemaphysalis punctata*, *Dermacentor reticulatus*, Disease ecology, Acari, Public health

## Abstract

**Background:**

South Downs National Park (SDNP) is UK’s most visited National Park, and a focus of tick-borne Lyme disease. The first presumed UK autochthonous cases of tick-borne encephalitis and babesiosis were recorded in 2019–20. SDNP aims to conserve wildlife and encourage recreation, so interventions are needed that reduce hazard without negatively affecting ecosystem health. To be successful these require knowledge of site hazards.

**Methods:**

British Deer Society members submitted ticks removed from deer. Key potential intervention sites were selected and six 50 m^2^ transects drag-sampled per site (mostly twice yearly for 2 years). Ticks were identified in-lab (sex, life stage, species), hazard measured as tick presence, density of ticks (all life stages, DOT), and density of nymphs (DON). Sites and habitat types were analysed for association with hazard. Distribution was mapped by combining our results with records from five other sources.

**Results:**

A total of 87 *Ixodes ricinus* (all but one adults, 82% F) were removed from 14 deer (10 *Dama dama*; three *Capreolus capreolus*; one not recorded; tick burden, 1–35) at 12 locations (commonly woodland). Five key potential intervention sites were identified and drag-sampled 2015–16, collecting 623 ticks (238 on-transects): 53.8% nymphs, 42.5% larvae, 3.7% adults (13 M, 10 F). Ticks were present on-transects at all sites: *I. ricinus* at three (The Mens (TM); Queen Elizabeth Country Park (QECP); Cowdray Estate (CE)), *Haemaphysalis punctata* at two (Seven Sisters Country Park (SSCP); Ditchling Beacon Nature Reserve (DBNR)). TM had the highest DOT at 30/300 m^2^ (DON = 30/300 m^2^), followed by QECP 22/300 m^2^ (12/300 m^2^), CE 8/300 m^2^ (6/300 m^2^), and SSCP 1/300 m^2^ (1/300 m^2^). For *I. ricinus*, nymphs predominated in spring, larvae in the second half of summer and early autumn. The overall ranking of site hazard held for DON and DOT from both seasonal sampling periods. DBNR was sampled 2016 only (one adult *H. punctata* collected). Woodland had significantly greater hazard than downland, but ticks were present at all downland sites. *I. ricinus* has been identified in 33/37 of SDNPs 10 km^2^ grid squares, *Ixodes hexagonus* 10/37, *H. punctata* 7/37, *Dermacentor reticulatus* 1/37.

**Conclusions:**

Mapping shows tick hazard broadly distributed across SDNP. *I. ricinus* was most common, but *H. punctata*’s seeming range expansion is concerning. Recommendations: management of small heavily visited high hazard plots (QECP); post-visit precaution signage (all sites); repellent impregnated clothing for deerstalkers; flock trials to control *H. punctata* (SSCP, DBNR). Further research at TM may contribute to knowledge on ecological dynamics underlying infection density and predator re-introduction/protection as public health interventions. Ecological research on *H. punctata* would aid control. SDNP Authority is ideally placed to link and champion policies to reduce hazard, whilst avoiding or reducing conflict between public health and ecosystem health.

## Introduction

### South Downs National Park

The South Downs National Park (SDNP) covers 1,627 km^2^ of the south-east of the British Isles, across Hampshire, West Sussex, and East Sussex. It encompasses two bioregions, a 140 km long chalk ridge called the South Downs, and a wooded lowland area called the Weald (from old English, meaning ‘forest’ (Hoad, 2003)). In total 23% is covered by woodland (SDNPA, 2019). Though much of the Park is subject to industrial agriculture, 4% remains species rich semi-natural chalk grassland, whilst 45% of its woodlands are legally classified as ancient (SDNPA, 2019) (*i.e*., wooded continuously since at least 1600 AD (Natural England & the Forestry Commission, 2022)). Both bioregions harbour biodiverse ecological communities of importance at national (*e.g*., 86 Special Areas of Scientific Interest (SSSIs)), and international scales (*e.g*., 13 Special Areas of Conservation (SACs)) (Crane & Williams, 2013). Portions of this text were previously published as part of a preprint: https://www.biorxiv.org/content/10.1101/2021.03.23.436533v1.full.

The Park is the most visited national park in the UK, with an estimated 39 million visitor days per year (NPUK, 2014), *c*. 120,000 people live and/or work within its borders, two million live within 5 km (SDNPA, 2020). UK national park designation does not indicate state or public ownership (unlike for example in the USA), and the great majority of land within national parks is privately owned (Butler, 2018). This is particularly so in the SDNP where 70% of the Park is farmed (SDNPA, 2019), and nearly a fifth (70,699 acres) is owned by just eight members of the gentry (two Dukes, three Viscounts, one Baron, and two Baronets) (Shrubsole, 2018, 2019). The public only have full statutory ‘Open Access’ to 4.4% of the Park (21,003 acres) (SDNPA, 2019). However, the area is crisscrossed by 3,218 km of public rights-of-way (TTC, 2018). Additionally, local authority owned country parks and land managed by the Forestry Commission (UK’s forest agency) bring a further 5% of the Park into public access (SDNPA, 2019), and some landowners also allow permissive paths. The South Downs Way, one of 15 UK national trails, is popular with walkers, cyclists, and horse-riders. In a 12-month period, 61,191 people passed one trail point (ESCC, 2016), locations close to carparks can be busier still (HCC, 2020).

### Ecology and epidemiology of tick-borne diseases

Ticks (Ixodida) are second only to mosquitoes globally as vectors of human pathogens (Lawrie et al., 2004). Twenty species of ticks are native to Great Britain (Jameson & Medlock, 2011), 26 to northwestern Europe as a whole (Hillyard, 1996). Most are relatively host specific and primarily nidicolous (*i.e*., living in or near shelters used by their hosts), and therefore of minimal risk to humans (Gray, Estrada-Pena & Vial, 2014). In contrast to nidicolous species, some ticks feed on diverse host communities, climbing undergrowth or litter to attach to passing vertebrates, including humans. One of these, *I. ricinus*, is the most common tick species across temperate Europe, is known to feed on over 300 wild or domestic vertabrates (Földvári, 2016), and is the tick most often affecting humans and pets in the UK (Jameson & Medlock, 2011; Abdullah et al., 2016; Davies et al., 2017). As a three-host tick, it blood feeds once from a different individual host at each active stage of its development, but spends most of its average 4–6 year life-cycle off-host (developing, in diapuse, or questing for a new host) (Hillyard, 1996; Földvári, 2016). Off-host habitat suitability is strongly influenced by descication; if litter has low humidity and/or high tempreture for a protracted period survival is threatened (Hillyard, 1996). Thus, across its range it is mainly associated with woodland, but in the British Isles especially it also inhabits humid valley pastures and upland rough grasslands (Kahl & Gray, 2023).

*Ixodes ricinus* host selection is partly determined by the height to which a tick climbs vegetation in quest of a host. In Britain, its larvae and nymphs feed primarily on rodents and birds (*e.g*., wood mice (*Apodemus sylvaticus*), grey squirrel (*Sciurus carolinensis*), blackbird (*Turdas merula*), pheasant (*Phasianus colchicus*), red grouse (*Lagopus lagopus scoticus*)) but can also be found on larger animals. Adults, which tend to climb higher, mainly parasitise larger mammals (*e.g*., sheep (*Ovis aries*), deer (*Capreolus capreolus*, *Cervus elaphus*, and *Dama dama*), and cattle (*Bos taurus*) (Craine, Randolph & Nutall, 1995; Hillyard, 1996; Kirby et al., 2004; Lihou & Wall, 2022). At each feed, engorgement can take between 2 and 11 days depending on the life stage: larvae, 2–5 days; nymphs, 2–7; adult females, 6–11 (Balashov, 1972, as cited in Földvári, 2016). Transovarial transmission of some pathogens can sometimes cause larvae to hatch as infectious (Hauck et al., 2020). However, a nymph feeding on a human will also have had opportunity to become infected when feeding as larva, and an adult will have already had two blood meals, potentially from very different animals. Thus *I. ricinus* can act as a vector for the transmission of pathogens to humans from diverse taxa (Hillyard, 1996; Randolph, 2009b; Mannelli et al., 2012). For example, it is the UK’s most common Lyme disease vector (Jameson & Medlock, 2011; Medlock & Leach, 2015). Dobson, Taylor & Randolph (2011) found *I. ricinus* nymphal questing in recreational areas of Southern England peaked in most habitats in spring between April and June. During this period all life-stages are active, but *I. ricinus* questing then tends to reduce during the hottest part of English summers (to avoid desiccation), with a resurgence in the latter part of summer and early autumn (UKHSA, 2023; NHM, 2024). Questing continues through the remainder of the year, but at a significantly reduced level (UKHSA, 2023).

In three regions in England and Wales, patients consulted general practitioners about tick-bites at a rate of 54–204 per 100,000 inhabitants in 2011, 72.5% of respondents in Cumbria had removed ticks from patients 2011–13 (101/100,000 population) (Gillingham et al., 2020). This is only a partial glimpse of the full extent of bites. An estimated ⅓–⅔ of tick bites go unnoticed (Hofhuis et al., 2015), and this can be particularly so with bites by larvae and nymphs, which are smaller than adults. Even if noticed many do not seek medical advice. For example, a 2007 population survey in the Netherlands found a tick bite incidence of 7,198/100,000, *c*. 1.1 million bites were reported. This equates to approximately fifteen times the number of tick-bite related general practice consultations (Hofhuis et al., 2015). Lyme disease is the primary human tick-borne disease of concern in the UK. Cairns et al. (2019) used general practice data to estimate a 1-year Lyme disease incidence of 12/100,000 (cautious interpretation is warranted, 59% of these clinical diagnoses lacked documented laboratory confirmation). Besides the causative agent of Lyme disease, *Borrelia burgdorferi* s.l., other human pathogens have been detected in ticks and related hosts in the British Isles, including: spotted fever group rickettsia (Tijsse-Klasen et al., 2011, 2013), *Borrelia miyamotoi* (Hansford et al., 2015), tick-borne encephalitis virus (Holding, Dowall & Hewson, 2020), and *Babesia venatorum* (Gray et al., 2019; Weir et al., 2020). In 2019–20 the first presumed autochthonous human cases of tick-borne encephalitis and babesiosis were recorded in the UK (PHE, 2020a). Some of these recently detected health threats may result from emerging foci of imported pathogens. However, it is also possible that in addition to Lyme disease, there may be considerable levels of undiagnosed tick-borne infections affecting persons in the British Isles.

Public Health England have mapped UK tick distributions at 10 km^2^ resolution by combining historical records (Pietzsch et al., 2005) with samples sent by the public, who most commonly found them on themselves, other human hosts, or their domestic pets, and to a lesser extent on livestock, and wild mammals and birds (Jameson & Medlock, 2011). UK general practice records of arthropod bites do not identify by species (Newitt et al., 2016), and though Hospital Episode Statistics (HES) have been used to map Lyme disease distribution (Cooper et al., 2017), HES records residential postcodes of patients, not where they were bitten. Thus, whilst HES is valuable to understanding disease burden, given UK tick-borne infections are very often linked to recreational exposure (Dobson, Taylor & Randolph, 2011), it’s use to map differing geographic tick hazard is limited. This is especially true in places such as the SDNP, with high numbers of regional, national, and international visitors.

Throughout this article we use ‘tick hazard’ to refer to tick species that routinely parasitise humans. Beyond simple presence/absence, tick density (‘an absolute measure describing the number of ticks per area unit’ (Kahl et al., 2002)) is the most reliable metric of site tick hazard (Ostfeld, 2011). However, knowledge of tick density is by its nature restricted to the relatively small number of places in Britain actively field-sampled. One of the most used density metrics is density of nymphs (DON) (Han et al., 2021). As Braks et al. (2016) discuss, though this is often reported as a risk metric, it is better understood as a hazard proxy. Given, as they state, a ‘hazard may not lead to a risk’ in the absence of someone being exposed to the hazard, risk at its most basic is ‘exposure times hazard’. An understanding of tick-bite risk at a particular site will then involve both ecological determinants of tick hazard and social determinants of exposure (*e.g*., visitor numbers, extent of use of protective clothing, *etc*.). In addition to DON, some field studies also report the metric density of ticks (DOT), which is the combined density of all life-stages of ticks collected (*e.g*., larvae, nymphs, adults) (for an example, see Han et al., 2021).

### Tick hazard in the SDNP

The SDNP is a priority area for interventions that reduce tick-borne disease hazard whilst preserving ecosystem health. Prior to our study its downland section had been highlighted by the Health Protection Agency (part-precursor to Public Health England) as a ‘regional foci of Lyme borreliosis’ (HPA, 2012), whilst West Sussex was listed alongside the South Downs as one of 10 areas in England and Wales where Lyme disease infection was most frequent (HPA, 2010). Yet despite this and the Park’s very large visitor numbers, prior to our study no multi-site field sampling of tick hazard in the SDNP, or comparison of hazard between its key habitats, had been published. Elsewhere woodland has been linked to increased tick-borne disease hazard, specifically Lyme disease (Gray et al., 1998; Killilea et al., 2008; Takken, 2016; Van Gestel et al., 2021; Bourdin et al., 2023), though controversy remains over causal pathways (Levy, 2013). For example, research linking forest fragmentation to increased Lyme disease hazard (summarised best in Ostfeld (2011)) has been criticized by UK researchers (Randolph & Dobson, 2012). Sheep grazing supports vector populations in some UK grass uplands, and though not host competent for *Borrelia burgdorferi* s.l., sheep can support transmission cycles *via* tick co-feeding (Ogden, Nuttall & Randolph, 1997), and also host *Babesia venatorum* (Gray et al., 2019). However, compared to wildlife, the role of livestock in propagation of tick-borne diseases of human concern is under-researched (Stanek et al., 2012). Increased wildlife populations have been implicated elsewhere in rising incidence of tick-borne disease (*e.g*., Crimean-Congo haemorrhagic fever in Turkey (Randolph, 2009a); tick-borne encephalitis in East Europe (Randolph, 2009b)) setting up a potential conflict between biodiversity and human health. Given UK National Parks aim to enhance wildlife and encourage public enjoyment of the countryside (NPUK, 2017), such conflict would be problematic for the South Downs National Park Authority (SDNPA) and the local governments from which most of its members are drawn. However, its joint remit, bioregional framing, and coalition of stakeholder members makes it the ideal body to link and champion site-based and regional policies to reduce hazard, whilst avoiding or reducing conflict between public health and ecosystem health.

### Aims

Our overall project (*Tick-borne hazards in the SDNP and the potential for Planetary Health based interventions*) includes (1) mapping and fieldwork to better understand tick hazard across the SDNP, including crucially at key potential locations for future interventions, (2) a systematic review of proposed interventions to reduce site hazard of the most common tick-borne disease in Britain, Lyme disease, with a focus on those actions not expected to negatively affect ecosystem health. Here, we report on our mapping and fieldwork, information on our systematic review can be found in Middleton, Cooper & Rott (2016).

Study objectives:

- identify and describe potential key locations for future interventions;
- map distribution of tick hazard across the SDNP;
- determine tick hazard (species and density) at potential intervention sites; and
- analyse habitat associations with tick hazard in the SDNP.

## Materials and Methods

### Sites selection for drag-sampling and potential future interventions

**Figure 1:**
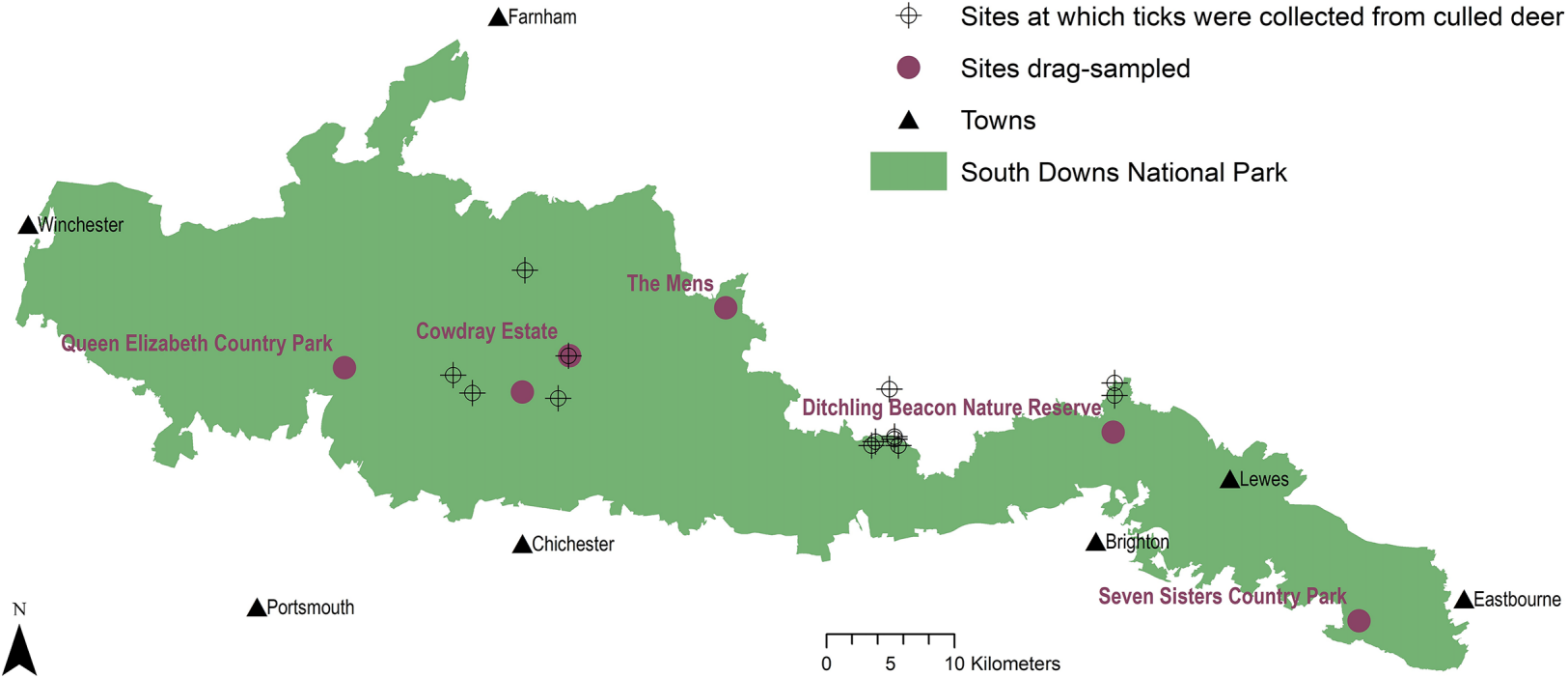
Tick sample collection sites in the South Downs National Park. Sites where ticks had been submitted by deerstalkers or drag-sampled by JM marked by points at 100 m^2^ resolution. All ticks collected from deer were *Ixodes ricinus*, which was also the only tick species drag-sampled at Queen Elizabeth Country Park, Cowdray Estate, and The Mens. *Haemaphysalis punctata* was the only tick drag-sampled at Seven Sisters Country Park and Ditchling Beacon Nature Reserve. Map contains OS data^©^ Crown Copyright (OS OpenData, 2020) and a National Park base layer (unmodified) from Natural England (2020) (https://creativecommons.org/licenses/by-nc-nd/2.0/). Map: JM.

Five sites were selected: three prospectively, and two responsively after submission of ticks obtained by deerstalkers from sentinel deer (Fig. 1). The three prospectively chosen sites were located one in each of the SDNP’s three counties. We took this approach so as to sample from along the National Park’s length, and because one of our project’s primary audiences is county authorities which manage countryside sites within the SDNP with high numbers of recreational visitors (*e.g*., UK accredited country parks as defined by NE and DEFRA (2014)). These authorities are key to implementing potential interventions to reduce tick-borne disease risks in the SDNP as they elect governing members to SDNPA (responsible for strategic action across SDNP), and directly manage downland and woodland sites with high visitor numbers where interventions could be trialled.

#### Prospectively selected

Of the three counties within SDNP’s borders, two county councils managed country parks in the National Park: Hampshire County Council (SDNP’s western section), and East Sussex County Council (SDNP’s eastern section). West Sussex County Council (SDNP’s central section) does not perform this function within the National Park. The SDNP’s ranger service was consulted about which Hampshire County Council and East Sussex County Council sites had the highest visitor numbers (subsequently confirmed by councils themselves).

**Figure 2:**
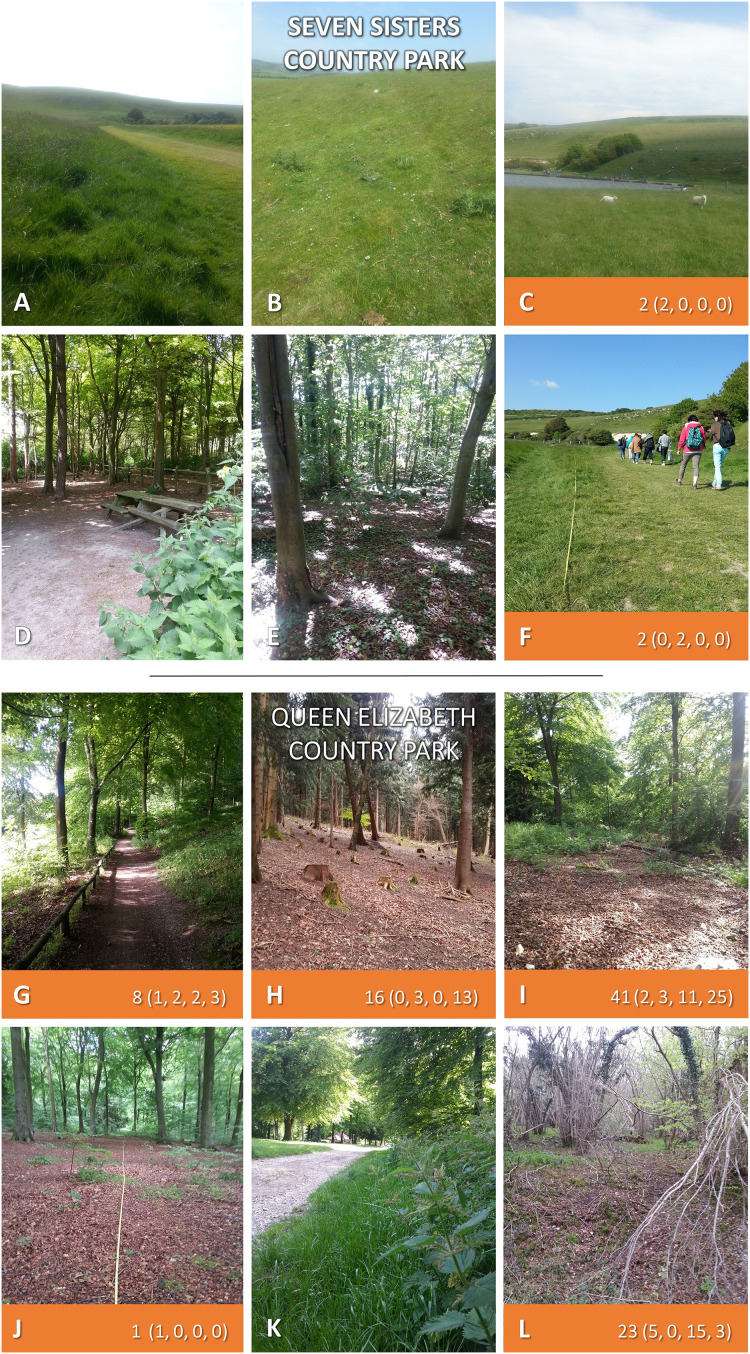
Seven Sisters Country Park, and Queen Elizabeth Country Park. Both sites sampled twice each in 2015 and 2016. Where ticks were present along 50 m^2^ transects 2-year totals are given (individual samplings in brackets). Photos: JM.

**Figure 3:**
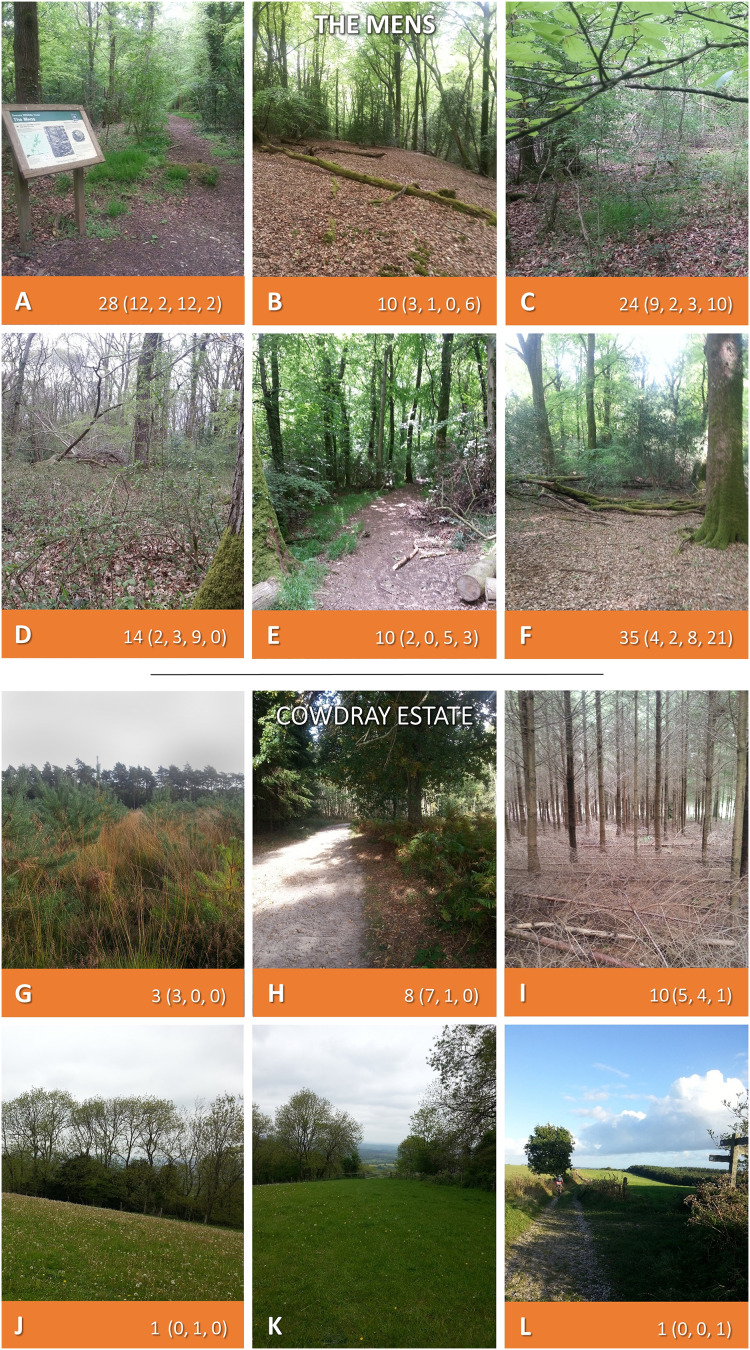
The Mens, and Cowdray Estate. Both sites sampled twice each in 2015 and 2016. Where ticks were present along 50 m^2^ transects 2-year totals are given (individual samplings in brackets). Photos: JM.

East Sussex County Council’s site with the highest annual visits (visitor details, and population and proximities of nearby towns, Table 1), was the 280 ha Seven Sisters Country Park (sevensisters.org.uk) in the SDNP’s eastern section. It is composed of chalk grassland, saltmarsh, shingle seashore, woodland, and a meandering river. Conservation designations include SSSI, Area of Outstanding Natural Beauty (AONB), Heritage Coast, and Marine Conservation Area. Transects (Figs. 2A–2F) consisted of sheep grazed chalk downland and woodland, primarily beech (*Fagus sylvatica*) and sycamore (*Acer pseudoplatanus)* (Table S1). Hampshire County Council’s site with the most visitors (Table 1) was the 564 ha Queen Elizabeth Country Park (https://www.hants.gov.uk/thingstodo/countryparks/qecp) in the SDNP’s western section. It consists of downland and wooded hills, designations include: SSSI, National Nature Reserve, Special Area for Conservation, Scheduled Ancient Monuments. All transects (Figs. 2G–2L) were in woodland (beech, conifer, or hazel (*Corylus avellana*)). Some had sparse undergrowth with dense beech or conifer litter, others nettle patches (*Urtica dioica*) or bramble thickets (*Rubus fruticosus* agg.) (Table S2). Given West Sussex County Council do not manage an appropriate site for sampling, a third site was chosen at SDNP’s centre which represented a sizeable wealden woodland owned by a key Park stakeholder (Sussex Wildlife Trust). The Mens is a 166 ha nature reserve of largely unmanaged wealden forest (sussexwildlifetrust.org.uk/visit/the-mens) (visitor details, Table 1). The site is especially rich in plants, saproxylic invertebrates, and fungi (*c*. 600 species). The sampled transects (Figs. 3A–3F) followed footpath borders tufted with grass, and cut across ground with sparse undergrowth under high canopies of predominantly beech, and sections with dense waist-high brambles (Table S3).

**Table 1:** Visitor information for sites drag-sampled.

Site	Nearest large populations	South Downs Way	Annual visitors	Carpark spaces (carparks, total m^2^)
Queen Elizabeth Country Park	Portsmouth: 19 km, est. 2019 pop. 229,851 (ONS, 2020).	Yes	202,559 vehicle entries March 2019–April 2020 recorded by number plate recognition system (HCC, 2020).	401 (8, 9,947)
Seven Sisters Country Park	Eastbourne: 8 km, est. 2019 pop. 114,809 (ONS, 2020). Brighton and Hove: 24 km, est. 2019 pop. 244,917 (ONS, 2020).	Yes	52,124 used visitor centre Jan–Dec 2019 (T. Wallace, 2020, personal communication), but only a minority of trips expected to include this.	292* (3, 6,542)
Ditchling Beacon Nature Reserve	Brighton and Hove: 4 km, est. 2019 pop. 244,917 (ONS, 2020).	Yes	Not recorded by land manager.	44* (2, 1,107)
Cowdray Estate	Chichester: 9 km, 2011 pop. 26,795 (ONS, 2015).	Yes	Not recorded by land manager.	10* (1, 315)
The Mens	Horsham: 18 km, 2011 pop. 49,000 (HDC, 2016).	No	Not recorded by land manager.	6* (1, 175)

**Note:**

* As only Queen Elizabeth Country Park (QECP) had individual marked-out car spaces in its carparks, space per m^2^ ratios from them were used to estimate capacity of each carpark at the other sites. In each case, the ratio chosen was from the QECP carpark closest in square metres to the unmarked site carpark (Article S1).

#### Responsively selected

The 6,677 ha Cowdray Estate is owned by Viscount Cowdray in the SDNP’s central section in West Sussex. It is a large private landholding with commercial deerstalking and mostly consists of forestry, downland, arable, and dairy/livestock farming. Transects (Figs. 3G–3L) sampled conifer plantation and sheep-grazed downland (Table S4). The final site (sampled 2016 only) was the 24 ha Ditchling Beacon Nature Reserve in East Sussex (sussexwildlifetrust.org.uk/visit/ditchling-beacon), managed by Sussex Wildlife Trust in the SDNP’s eastern section. It consists of downland plateau and steep scarp slopes of chalk grassland and woods. Parts of Ditchling Beacon Nature Reserve are under conservation grazing with sheep/cattle. The escarpment is an SSSI harbouring flower rich chalk grassland, rare orchids, and butterflies. Transects ran through grazed downland, some bordering hawthorn (*Crataegus monogyna*) and ash (*Fraxinus excelsior*) scrub, and along verges of footpaths leading from carparks.

### Tick collection from deer

Deerstalkers were recruited through the British Deer Society (bds.org.uk) newsletter and website, sent kits, and asked to collect ticks from deer culled for reasons unrelated to this project. Participants were instructed to inspect the whole animal, collect every visible tick, place them in pre-coded 1 ml cryovials (pre-filled with 0.5 ml 70% ethanol) and return by post (safety measures, Article S1). On receipt cryovials were deposited in a laboratory fridge (approx. 5 °C), and after identification transferred to a freezer (approx. −20 °C). Deerstalkers recorded: habitat type; deer species; six-figure grid reference (using ‘OS Locate’ (Ordnance Survey, London, 2014)); body sites ticks found at; and whether ticks were attached or not.

### Tick collection by drag-sampling

Sites were sampled April to November inclusive. To collect questing ticks, sampling was not carried out when air temperature was <7 °C 50 cm above the ground or when vegetation was wet from recent rain/dew, as per James et al. (2013). Four sites were sampled in both 2015 and 2016, with an additional site sampled in 2016. At each, six 1 m × 50 m transects were sampled as per Dobson, Taylor & Randolph (2011). The first two transects chosen were those suspected to have the highest potential exposure of humans to ticks (*e.g*., vegetation alongside a footpath). Where sites included grassland and woodland, one chosen transect was selected from each. All others were selected using dice and a random number table. To reduce spurious conclusions from single sampling, each transect was planned to be sampled twice yearly, for 2 years. Over the 2 years of fieldwork each of the four sites analysed for difference were sampled during a 58-day spring period (beginning 23^rd^ April and ending by close of UK astronomical spring around 21^st^ June), and a 59-day period in the second half of summer and very early autumn (starting 2^nd^ August, ending 29^th^ September). This sampling was not conducted in between as early to mid-summer includes July which is generally the hottest UK month (MO, 2024), and known to see less *I. ricinus* questing. Additionally, the spring period included UK May public holidays, whilst the second half of summer and very early autumn period included most of the UK school summer holiday. Thus, these two seasonal sampling periods are of particular importance for establishing UK tick hazard due to known peaks in both tick questing and people using the countryside for recreation. At one private site used for game shooting, it was not possible to visit twice in 2016 due to a requirement to be accompanied by a deerstalker with restricted availability, and this also delayed some sampling.

**Figure 4:**
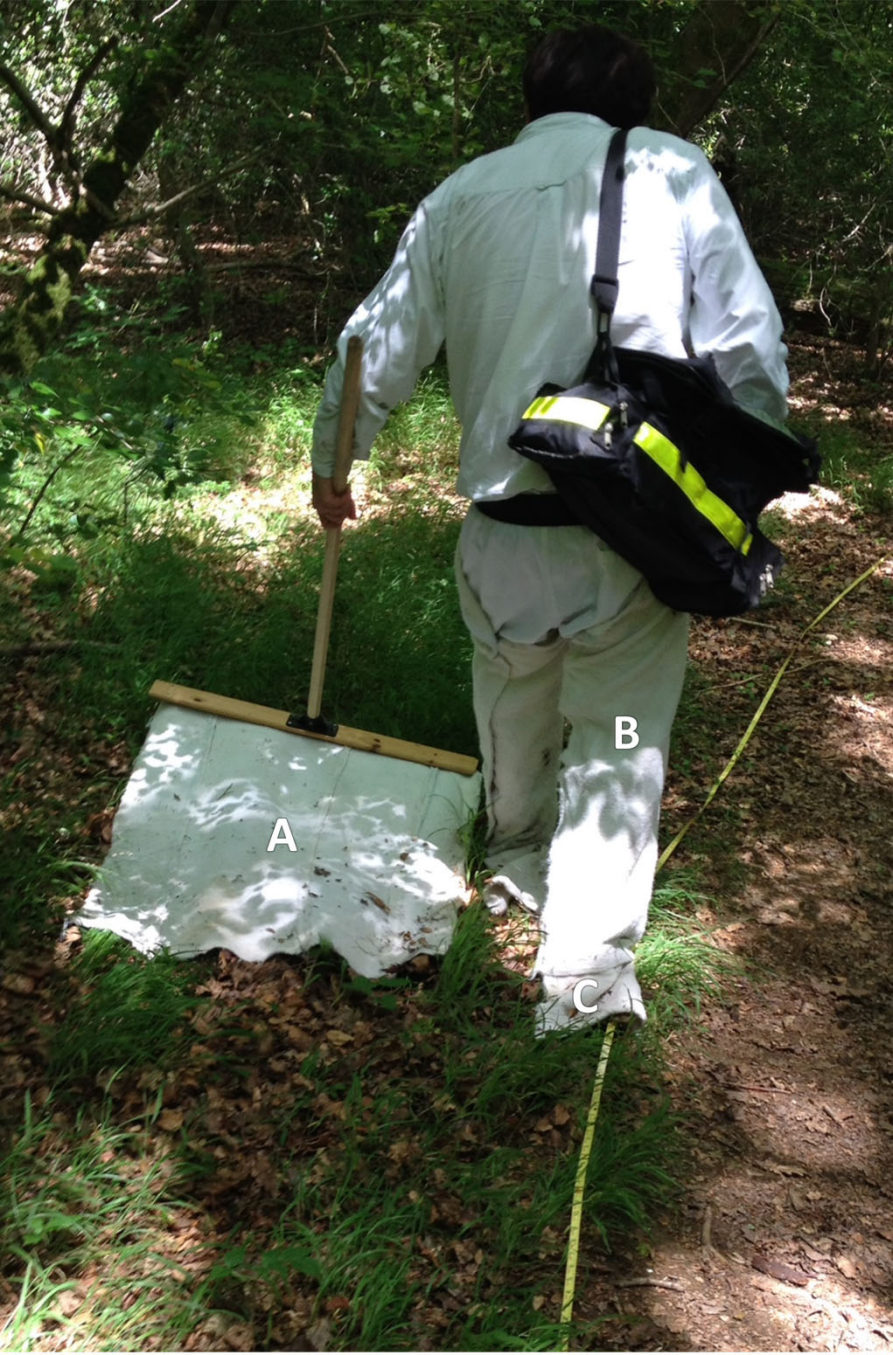
Site tick-sampling equipment. JM drag-sampling along a path border at The Mens, West Sussex. (A) Woollen blanket. (B) Woollen chaps. (C) Woollen flags. Design as per Dobson, Taylor & Randolph (2011). Photo: ASR.

To improve chances of picking up disease signals in planned follow-up research (usually only a minority of ticks at any site are infected (Vollmer et al., 2011)), extras were acquired by drag-sampling between transects, and at follow up visits where possible. Tick sampling techniques differ in efficacy and are affected by habitat/vegetation type (Dantas-Torres et al., 2013). To reduce bias ticks were collected simultaneously along transects using a blanket drag, heel flags, and chaps (all made from wool, Fig. 4). Wools were examined after each transect, ticks placed individually in 70% ethanol filled micro-centrifuge tubes, deposited same day in a laboratory freezer (approx. −20 °C). At each transect, at each sampling, photos were taken along with field notes including: ticks collected; date/time; weather; habitat; visitors observed; dominant vegetation; vegetation height; main litter constituents; relative humidity and temperature (both at 50 cm and in litter, measured with a Fisher Scientific Traceable Hygrometer). Locations were recorded (10-figure OS grid references, bearings) using ‘OS Locate’ and ‘OS Mapfinder’ (Ordnance Survey, London, 2014) on a Samsung Galaxy Note II phone (Researcher safety and inter-site contamination control, Article S1).

### Tick identification

Identification was conducted in-lab with a hand lens (Hilkinson Ruper x20 15 mm achromatic), and where necessary a dissecting microscope (Leica EZ4; Wetzlar, Germany). A species key was used (Hillyard, 1996), and identification aided by reference to Baker (1999) and Beati, Needham & Klompen (2016). Species and life stage was recorded, adult ticks sexed. Larvae were not fully keyed as clearing for slide mounting would have reduced the sample pool available for future pathogen detection. However, each larva was inspected for characters which identified them to genus, and indicated likely species. If nymphs/adults of more than one species were identified at any site, 10% of larvae from that site (to a maximum of 50) would have been slide mounted and keyed. A limitation of many similar studies has been not enabling retrospective evaluation of species identification (Estrada-Peña et al., 2013). To corroborate identification, voucher specimens (including all life stages/sexes) have been deposited into the University of Oxford Museum of Natural History Arachnida Collection (Accession Lot No. ENT-OUMNH-2024-009).

### Mapping distribution of tick hazard

Using ArcMap 10.7 (ESRI, Redlands, CA, USA) sites where ticks had been submitted by deerstalkers or drag-sampled by JM were mapped, indicated by points at 100 m^2^ resolution. In addition, to map recorded presence of tick hazard at 10 km^2^ resolution by species, data from the following sources were compared and combined as layers: (1) the most recent published Public Health England/Health Protection Agency tick maps for England and Wales (Cull et al., 2018; PHE, 2016; HPA, 2013a, 2013b, 2013c, 2013d) (2) National Biodiversity Network Atlas (NBN, 2020) (which includes historic data 1890 onwards, and into which Public Health England now submits tick records (PHE, 2020b)), (3) a single site drag-sample in 2014 by Layzell et al. (2018), (4) point locations of ticks submitted from culled deer or collected by drag-sampling in this project 2015–16, (5) point locations drag-sampled for *Haemaphysalis punctata* by Public Health England and the Animal and Plant Health Agency, primarily 2015–18 (Medlock et al., 2018), (6) records from pan-species surveying at Sussex Wildlife Trust reserves, mainly 2016–17 (previously unpublished data collected by Trust volunteers and staff and supplied directly to the authors for inclusion in this study). Digital basemaps were obtained from OS OpenData (2020) and Natural England (2020). (Layer generation detailed in Article S1).

### Site visitors

In addition to receiving guidance from rangers and local authorities on which sites they managed in the SDNP had the highest amount of visitors, land managers were asked for annual site visitor numbers when available. Population sizes of nearby towns were taken from reports of the UK Office for National Statistics (ONS). All five drag-sampled sites were rural, and most visitors annually could be expected to arrive by private vehicles. As Weitowitz et al. (2019) demonstrated in their nationwide survey of parking provision at UK nature conservation sites, car park capacity is broadly predictive of differences in visitor numbers. Thus, to indicate relative difference in number of expected site visitors (and thus exposure to site tick hazard), all car parks (15 in all) at each of the five sites were measured on 25 December 2023. Car space per m^2^ ratios from Queen Elizabeth Country Park’s eight carparks (the only ones with marked-out individual car spaces, Article S1) were then used to estimate number of carpark spaces at the other sites (Table 1). No changes to the carparks had been made in the period between the tick survey and carpark measurements.

### Analysis

As well as site vector presence/absence, for sites drag-sampled both years tick hazard was assessed as (1) questing density of ticks, all life stages (DOT), and (2) questing density of nymphs (DON). These were calculated as means of totals of four site samplings: six 1 m × 50 m transects, sampled twice yearly for 2 years. Additionally, DOTs and DONs were calculated for each of the two seasonal sampling periods. To determine significance of difference of tick (all life stages) and nymph counts between the four sites sampled in both years, Kruskal-Wallis Tests with Bonferroni corrected *post-hoc* Dunn’s Tests were carried out on counts from all individual transect samplings (*i.e*., 90 50 m × 1 m drag-samplings). Further Kruskal-Wallis Tests with Bonferroni corrected *post-hoc* Dunn’s Tests examined habitat types determining tick (all life stages) and nymph hazard. Statistical analysis was carried out in Minitab17 (Minitab Inc, State College, PA, USA). Drag-sampling results from the additional site sampled in 2016 are reported as presence/absence, and in distribution mapping, but were not included otherwise in analysis (justification of analysis, Article S1).

## Results

Figure 1 maps locations ticks were submitted from by deerstalkers, and five potential future intervention sites drag-sampled across the SDNP by the first author. Ecological descriptions are given for the five sites in the materials and methods section, annual visitor numbers (where available), estimated carpark spaces, and locations and populations of nearby towns are provided in Table 1.

### Extent of tick hazard across the SDNP

#### Distribution and species

**Figure 5:**
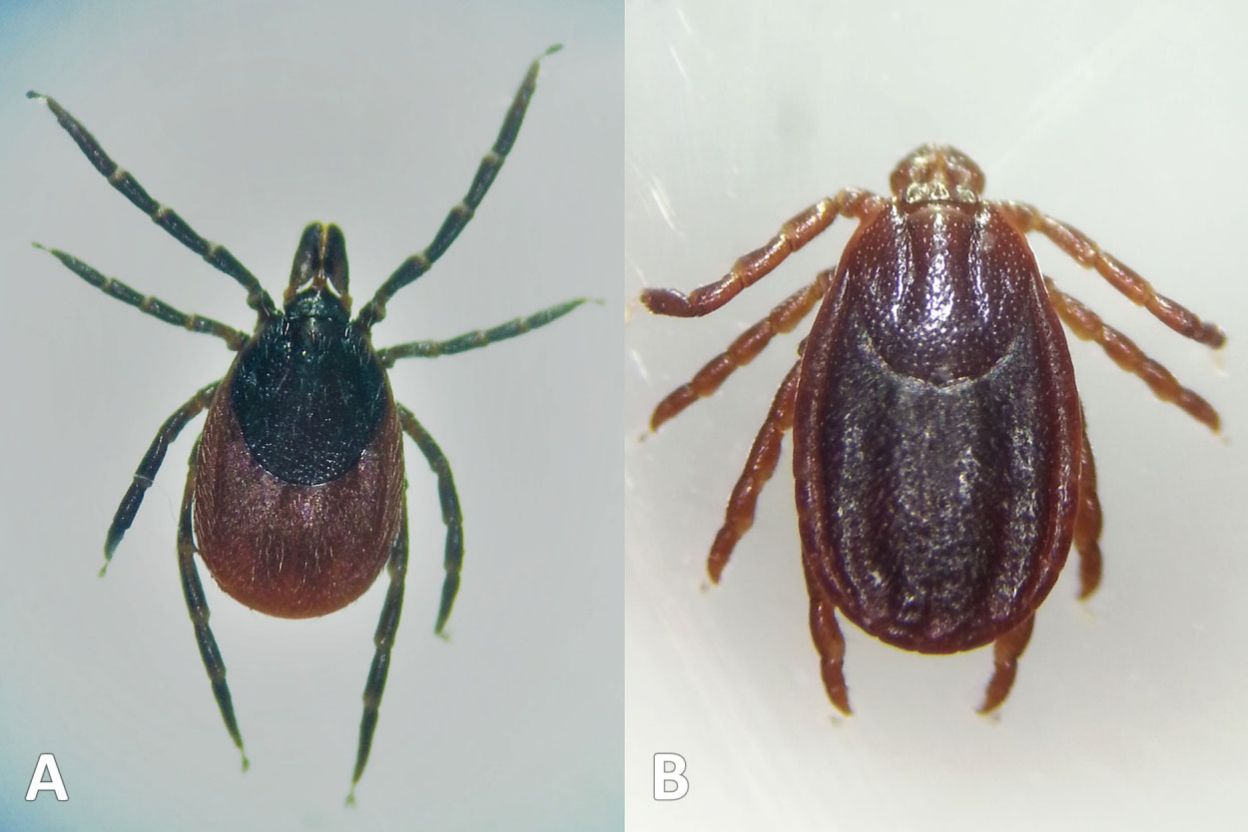
Tick species collected. Ticks collected during study. (A) *Ixodes ricinus* (drag-sampled at The Mens, West Sussex, 2016). (B) *Haemaphysalis punctata* (drag-sampled at Ditchling Beacon Nature Reserve, East Sussex, 2016). Photos: JM.

Ticks collected by drag-sampling or submitted by deerstalkers confirmed presence across much of SDNP (Fig. 1). Ticks were present in both of its characteristic habitats: sheep grazed downland, and wealden woods. Ticks were found at all sites drag-sampled (though not on all transects, Figs. 2 and 3; Tables S1–S4). All ticks submitted from deer were *Ixodes ricinus* (Fig. 5A), also the only species collected at three of the four sites drag-sampled in both 2015 and 2016 (Queen Elizabeth Country Park, The Mens, Cowdray Estate). The nationally rare *H. punctata* (Fig. 5B) was the sole tick collected from the remaining site sampled in both years (Seven Sisters Country Park), and was also found at Ditchling Beacon Nature Reserve in 2016.

**Figure 6:**
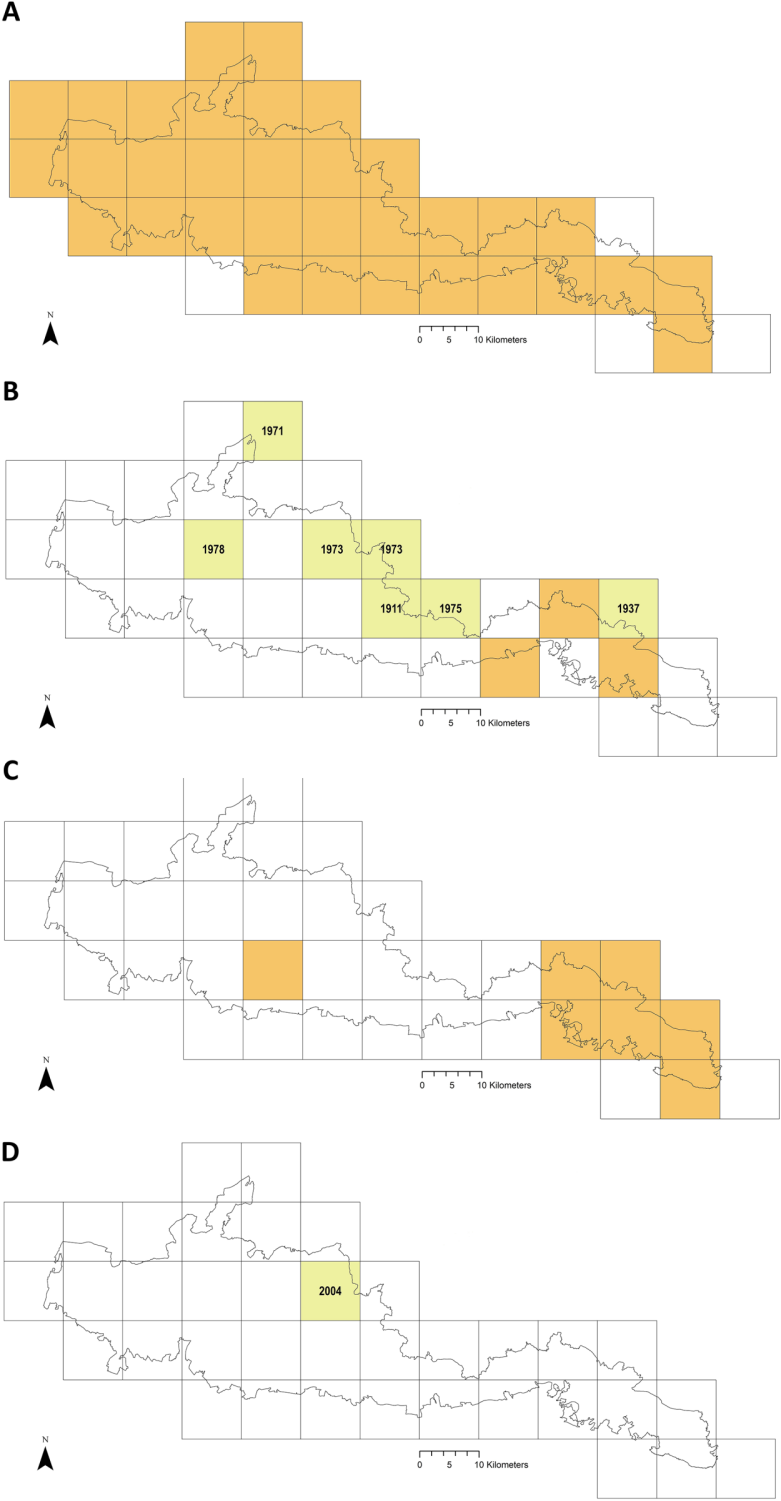
Recorded tick hazard in the South Downs National Park. (A) *Ixodes ricinus*. (B) *Ixodes hexagonus*. (C) *Haemaphysalis punctata*. (D) *Dermacentor reticulatus*. Orange OS grid squares indicate the most recent record/s of tick presence are since 2005 (inclusive). Yellow squares indicate the most recent record/s found were prior to 2005 (latest record date shown). Empty squares represent no records found, but should not be taken as on-the-ground tick absence. Map combines our data of drag-sampling and ticks submitted from culled deer, national maps from the Public Health England/Health Protection Agency tick surveillance scheme (Cull et al., 2018; PHE, 2016; HPA, 2013a, 2013b, 2013c, 2013d), the National Biodiversity Network Atlas NBN (2020), Medlock et al. (2018), Layzell et al. (2018), and previously unpublished records from pan-species surveying at Sussex Wildlife Trust reserves (collected by Trust volunteers and staff and supplied directly to the authors for inclusion in this study). In addition, a case report by Phipps et al. (2020) states there were *H. punctata* infestations within the confines of Brighton & Hove in 2019. Maps contain OS data^©^ Crown Copyright (OS OpenData, 2020) and a National Park base layer (unmodified) from Natural England (2020) (https://creativecommons.org/licenses/by-nc-nd/2.0/). Maps: JM.

Figure 6 maps distribution of tick records at 10 km^2^ resolution. The first report for *I. ricinus* is from 1964, 33/37 of the Park’s grid squares have had at least one record (often multiple) in the last 15 years (Fig. 6A). In contrast, *Ixodes hexagonus* (the UK’s second most common Lyme disease vector (Jameson & Medlock, 2011; Medlock & Leach, 2015) has been recorded far less, most squares where presence has been recorded represent historic records only (Fig. 6B). The earliest *H. punctata* report is from 1920, but all related grid squares have had recorded presence in the last decade, mostly in its known foci in the far east of the Park (Fig. 6C). Locations included from recent drag-sampling by Public Health England and Animal and Plant Health Agency suggests it has spread westwards somewhat, and this observation by Medlock et al. (2018) is confirmed by drag-sampling in our study at Ditchling Beacon Nature Reserve which extends its known range further still, as does a previously unpublished isolated recording by Sussex Wildlife Trust 44 km further west. A second rare species in the UK, *Dermacentor reticulatus*, was recorded by Sussex Wildlife Trust at one of its West Sussex reserves in 2004 (Fig. 6D). To our knowledge *D. reticulatus* has not otherwise been recorded in the SDNP or its constituent counties; this record was not previously included in National Biodiversity Network Atlas or Public Health England/Health Protection Agency published mapping.

#### Ticks collected from deer

**Figure 7:**
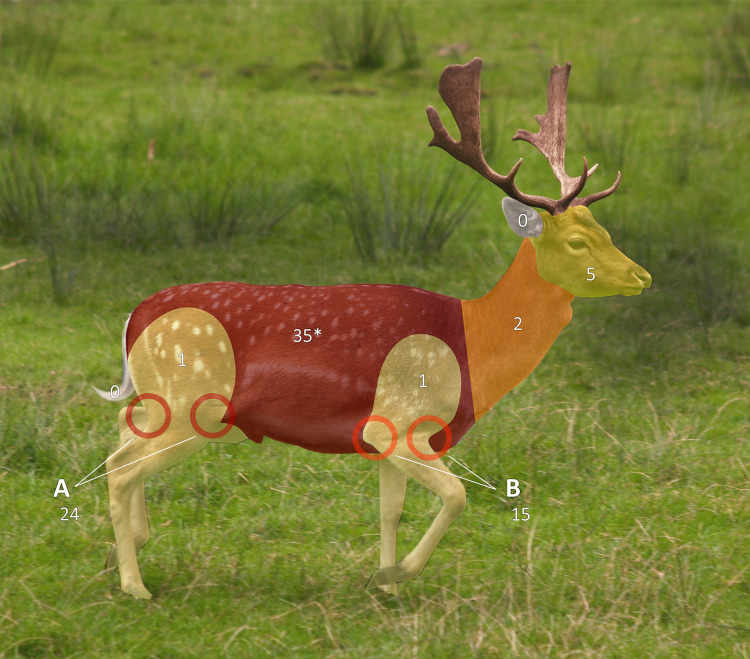
Tick attachment sites on sentinel deer. Ticks collected by British Deer Society members from deer culled for other reasons, attachment sites for four ticks not recorded. Body zones as per Pacilly et al. (2014). *Instructions listed abdomen and sternum as separate zones to record, but for 10 ticks this was not done so zones were merged in this figure (reported attachment sites: abdomen, 24; sternum and abdomen, 10; sternum, 1). Photo: Andreae (2008), use and changes made under CC-BY-SA-2.0 which also applies to this figure. Original: https://web.archive.org/web/20200930080151/https://commons.wikimedia.org/wiki/File:Fallow_deer_in_field_%28cropped%29.jpg.

Eighty-seven ticks were submitted (Table 2) obtained from 14 deer at 12 locations (Fig. 1). All but one were adult ticks, and 82% of those were females. Most males were attached in mating; the majority of females were engorged (73%, *n* = 71, two not recorded by collectors), as was the sole nymph. The commonest habitat ticks were collected off deer at was ‘wood’ (59%, *n* = 87). Most of the remaining were from deer shot at mixed edge-habitats involving wood (28%, *n* = 87), *e.g*. ‘wood-heath’. Most hosts were fallow deer (*Dama dama*) (10, 71%), followed by roe (*Capreolus capreolus*) (3, 21%) (*n* = 14, one host species not recorded by collector). Tick burden was 1*–*35 per deer. The commonest attachment sites were abdomen and sternum, and posterior and frontal axillae (Fig. 7).

**Table 2:** Ticks submitted by deerstalkers.

*n* = 87		Tick (%)
Tick species		
*Ixodes ricinus*		87 (100)
Tick life stage and sex		
Adult females		71 (82)
Adult males		15 (17)
Nymph		1 (1)
Larvae		0 (0)
Female engorgement (*n* = 71)		
Females engorged*		52 (73)
Females not engorged		19 (27)
Not recorded		2 (3)
Habitat		
Wood		51 (59)
Wood-chalk grassland		15 (17)
Wood-pasture		7 (8)
Pasture		6 (7)
Wood-arable		3 (3)
Wood-heath		3 (3)
Other		2 (2)
Host species		
Fallow deer, *Dama dama* (*n* = 10)		45 (52)
Roe deer, *Capreolus capreolus* (*n* = 3)		38 (44)
Not recorded (host *n* = 1)		4 (5)
Range of ticks per host		1–35

**Notes:**

Deer culled for reasons unrelated to this project. Percentages rounded to whole numbers. Engorged status covers adult females and nymphs only, as adult males do not engorge.

* in addition the single nymph was engorged.

#### Ticks collected by drag-sampling

**Figure 8:**
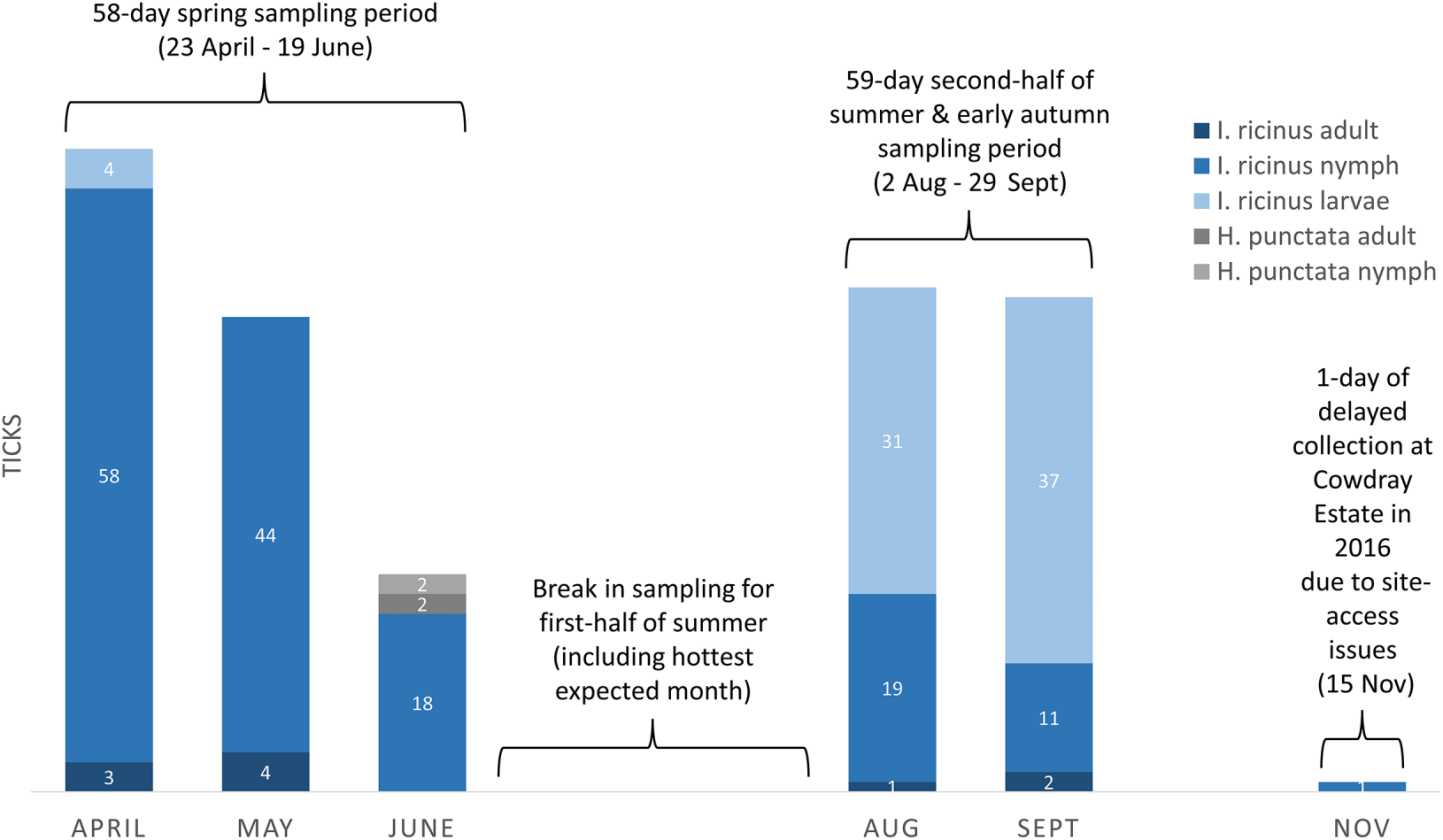
Ticks collected on-transect (by life stage, species, month, and seasonal sampling period) from sites in the South Downs National Park drag-sampled both 2015 and 2016. Densities of nymphs and densities of ticks (all life stages) are provided in the main text for each site, calculated by seasonal sampling periods, and on overall multi-year basis. Number of ticks obtained along transects at each sampling, collection dates and environmental data, Tables S1–S4.

Drag-sampling four sites in both 2015 and 2016 collected 622 ticks (Table 3). Of these, 237 were along transects and are included in calculations of vector densities and analysed statistically. A total of 385 extras were stockpiled to aid future pathogen detection. Ticks were present at all four sites and the additional site sampled twice in 2016 (one adult only). Of ticks collected by drag-sampling at all sites (*n* = 623), most were nymphs (53.8%), followed by larvae (42.5%), and a small number of adults (3.7%, 13 males, 10 females (Tables S1–S4). 93.3% (222) of ticks gathered along transects (*n* = 238) had attached to woollen blankets, 6.7% (16) to woollen chaps, and none were attached to flags (Table 3). Figure 8 shows a breakdown of tick data from transects by collection month, and by tick life stage and species (all sites sampled in both 2015 and 2016, *n* = 237). Of these, *H. punctata* only made up a small number (1.7%, four; two nymphs and two adults, both collected in the spring sampling period), whilst *I. ricinus* made up the vast majority (233, 98.3%). Most of these *I. ricinus* were nymphs (64.8%, 120 from spring, 30 from the late summer and early autumn sampling period). In addition, 72 (30.9%) were larvae (spring, four; late summer early autumn, 68), and 10 (4.3%) were adults (spring, seven; late summer early autumn, three).

**Table 3:** Ticks collected by site drag sampling.

			On-transect				Off-transect				Site totals
			Life stages and sex				Life stages and sex				
	Species	Collected on	Larvae	Nymphs	Adults	Totals	Larvae	Nymphs	Adults	Totals*	
Seven Sisters Country Park Six 50 m^2^ transects, each sampled four times (twice each in 2015 & 2016).	*H. punctata*	Blanket		2		4	65	8	1♀	74	78
		Chaps			2♀						
		Flags									
Queen Elizabeth Country Park Six 50 m^2^ transects, each sampled four times (twice each in 2015 & 2016).	*I. ricinus*	Blanket	37	39	1♀ 4♂	89	25	65	3♀ 1♂	94	183
		Chaps		8							
		Flags									
The Mens Six 50 m^2^ transects, each sampled four times (twice each in 2015 & 2016).	*I. ricinus*	Blanket	35	78	1♀ 3♂	121	100	104	2♀ 3♂	209	330
		Chaps		4							
		Flags									
Cowdray Estate Six 50 m^2^ transects, each sampled three times (twice in 2015, once in 2016).	*I. ricinus*	Blanket		21	1♂	23	3	5		8	31
		Chaps		1							
		Flags									
Ditchling Beacon Nature Reserve Six 50 m^2^ transects, each sampled two times (twice in 2016).	*H. punctata*	Blanket			1♂	1				0	1
		Chaps									
		Flags									
		Totals	72	153	13	238	193	182	10	385	623

**Note:**

Results of individual samplings in Supplemental Material, Tables S1–S4.

* Off-transect ticks were those additionally collected opportunistically for later pathogen detection, and were not included in calculations of DON or DOT.

### Tick hazard at potential sites for interventions

Figures 2 and 3 and Tables S1–S4 show the number of ticks obtained along transects at each sampling (the latter also provide collection dates and environmental data).

#### Sites at which *H. punctata* were collected

Seven Sisters Country Park (Figs. 2A–2F; Table S1): 78 *H. punctata* ticks were collected (four along transects), representing all life stages. All ticks were collected on downland, none on the wooded transects (Table S1). Tick hazard ranged 0–2 ticks per 50 m^2^ individual sampling, site DON and DOT were both 1 per 300 m^2^ (Table 4A). The area which provided most off-transect ticks (*i.e*., those additionally collected opportunistically for later pathogen detection, and not included in calculation of DON or DOT) is in the background of the photo of transect 3 (Fig. 2C), hosting a school picnic. The combined multi-year spring DON = 0.2 per 50 m^2^ (range 0–2), DOT=0.3 per 50 m^2^ (0–2). The second half of summer and early autumn DON = 0.0 per 50 m^2^ (0–0), DOT=0.0 per 50 m^2^ (0–0).

**Table 4:** Site and habitat tick hazard, with Kruskal-Wallis and Bonferroni corrected *post-hoc* Dunn’s tests of nymphs and ticks (all life stages).

(A) Tick hazard at drag-sampled sites					
	Tick species	Ticks found on transects	Ticks per 50 m ^2^ sampling	Site density of nymphs (DON)	Site density of ticks (DOT)
The Mens	*I. ricinus*	121	0–21	30/300 m^2^	30/300 m^2^
Queen Elizabeth Country Park	*I. ricinus*	89	0–25	12/300 m^2^	22/300 m^2^
Cowdray Estate	*I. ricinus*	23	0–7	6/300 m^2^	8/300 m^2^
Seven Sisters Country Park	*H. punctata*	4	0–2	1/300 m^2^	1/300 m^2^
Ditchling Beacon*	*H. punctata*	1	0–1	n/a	n/a

(B) Site comparison between ticks collected on transects		
Sites	Nymphs	Ticks (all life stages)
The Mens	10, 2, 12, 1, 3, 1, 0, 2, 8, 2, 3, 4, 2, 3, 6, 0, 2, 0, 3, 2, 4, 2, 8, 2	12, 2, 12, 2, 3, 1, 0, 6, 9, 2, 3, 10, 2, 3, 9, 0, 2, 0, 5, 3, 4, 2, 8, 21
Seven Sisters Country Park	0, 0, 0, 0, 0, 0, 0, 0, 0, 0, 0, 0, 0, 0, 0, 0, 2, 0, 0, 0, 0, 0, 0, 0	0, 0, 0, 0, 0, 0, 0, 0, 0, 0, 0, 0, 0, 0, 0, 0, 2, 0, 0, 0, 0, 2, 0, 0
Queen Elizabeth Country Park	1, 1, 2, 3, 2, 2, 10, 1, 1, 0, 0, 0, 0, 0, 0, 0, 5, 0, 14, 1, 0, 1, 0, 3	1, 2, 2, 3, 2, 3, 11, 25, 1, 0, 0, 0, 0, 0, 0, 0, 5, 0, 15, 3, 0, 3, 0, 13
Cowdray Estate	3, 0, 0, 7, 1, 0, 5, 4, 1, 0, 0, 0, 0, 0, 0, 0, 0, 1	3, 0, 0, 7, 1, 0, 5, 4, 1, 1, 0, 0, 0, 0, 0, 0, 0, 1
**Results of Kruskal-Wallis tests**	H = 31.59 (DF 3, *n* = 90) ***p* = 0.000** (adjusted for ties)	H = 31.93 (DF 3, *n* = 90) ***p* = 0.000** (adjusted for ties)

**Bonferroni corrected *post-hoc* Dunn’s Tests†** ** (Bonferroni Z-value = 2.128)**	*Z*	*p*	*Z*	*p*
The Mens *vs* Cowdray Estate	3.46104	**0.00054**	3.22229	**0.00127**
The Mens *vs* Seven Sisters Country Park	5.48936	**0.00000**	5.50030	**0.00000**
The Mens *vs* Queen Elizabeth Country Park	2.50985	**0.01208**	2.08591	0.03699
Cowdray Estate *vs* Seven Sisters Country Park	1.62112	0.10499	1.87000	0.06148
Cowdray Estate *vs* Queen Elizabeth Country Park	1.13737	0.25538	1.29111	0.19667
Seven Sisters Country Park *vs* Queen Elizabeth Country Park	2.97951	**0.00289**	3.41439	**0.00064**

(C) Habitat comparison between ticks collected on transects		
Habitats	Nymphs	Ticks (all life stages)
Deciduous woodland	10, 2, 12, 1, 3, 1, 0, 2, 8, 2, 3, 4, 2, 3, 6, 0, 2, 0, 3, 2, 4, 2, 8, 2, 0, 0, 0, 0, 0, 0, 0, 0, 1, 1, 2, 3, 2, 2, 10, 1, 1, 0, 0, 0, 0, 0, 0, 0, 5, 0, 14, 1	12, 2, 12, 2, 3, 1, 0, 6, 9, 2, 3, 10, 2, 3, 9, 0, 2, 0, 5, 3, 4, 2, 8, 21, 0, 0, 0, 0, 0, 0, 0, 0, 1, 2, 2, 3, 2, 3, 11, 25, 1, 0, 0, 0, 0, 0, 0, 0, 5, 0, 15, 3
Conifer woodland/planting	3, 0, 0, 7, 1, 0, 5, 4, 1, 0, 1, 0, 3	3, 0, 0, 7, 1, 0, 5, 4, 1, 0, 3, 0, 13
Downland	0, 0, 0, 0, 0, 0, 0, 0, 1, 0, 0, 0, 0, 0, 0, 0, 0, 2, 0, 0, 0, 0, 0, 0, 0	1, 0, 0, 0, 0, 0, 0, 0, 1, 0, 0, 0, 0, 0, 0, 0, 0, 2, 0, 0, 0, 0, 2, 0, 0
**Results of Kruskal-Wallis tests**	H = 19.95 (DF 2, *n* = 90) ***p* = 0.000** (adjusted for ties)	H = 18.10 (DF 2, *n* = 90) ***p* = 0.000** (adjusted for ties)

**Bonferroni corrected *post-hoc* Dunn’s Tests‡ (Bonferroni Z-value = 1.834)**	*Z*	*p*	*Z*	*p*
Deciduous woodland *vs* Conifer woodland/planting	0.16709	0.86730	0.19196	0.84777
Deciduous woodland *vs* Downland	4.35820	**0.00001**	4.15870	**0.00003**
Conifer woodland/planting *vs* Downland	2.95039	**0.00317**	2.78585	**0.00534**

**Notes:**

Detailed results of individual samplings (including collection dates and environmental data) in Supplemental Material, Tables S1–S4. Bolded *p*-values from *Post-hoc* Dunn’s Tests were significant differences (<0.05), with Z values >= the Bonferroni Z-value (sign CIs and magnitudes/directions of differences, Figs. S1 and S2).

* Ditchling Beacon was drag-sampled in 2016 only (following deerstalker tick submission), so not included in calculation of DON, DOT, or site and habitat comparisons. All other sites drag-sampled both 2015 and 2016.

† Nymphs: comparisons, 6; ties, 78; family alpha, 0.2; Bonferroni individual alpha, 0.033. Ticks (all life stages): comparisons, 6; ties, 73; family alpha, 0.2; Bonferroni individual alpha, 0.033.

‡ Nymphs: comparisons, 3; ties, 78; family alpha, 0.2; Bonferroni individual alpha, 0.067. Ticks (all life stages): comparisons, 3; ties, 73; family alpha, 0.2; Bonferroni individual alpha, 0.067.

Ditchling Beacon Nature Reserve: One single *H. punctata* tick was collected on-transect on semi-grazed downland adjacent to scrub woodland, none on the other five transects at either set of samplings in 2016. The adult male tick had attached to the blanket (Table 3). Densities were not calculated due to 1-year only sampling (Article S1).

#### Sites at which *I. ricinus* were collected

Queen Elizabeth Country Park (Figs. 2G–2L; Table S2): 183 *I. ricinus* ticks were collected (89 on-transect), from all life stages. Ticks were found on five of the six transects, all wooded or on paths verging woodland (Table S2), range 0–25 per 50 m^2^ sampling, DON=12 per 300 m^2^, DOT=22 per 300 m^2^ (Table 4A). Spring DON=3.2 per 50 m^2^ (range 1–14), DOT=3.4 per 50 m^2^ (0–15). Second half of summer and early autumn DON=1.2 per 50 m^2^ (0–5), DOT=3.9 per 50 m^2^ (0–25).

The Mens (Figs. 3A–3F; Table S3): 330 *I. ricinus* ticks were collected (121 on-transect), representing all life stages. Ticks were obtained from all six transects (Table S3), range 0–21 per 50 m^2^ sampling, DON=21 per 300 m^2^, DOT=30 per 300 m^2^ (Table 4A). Spring DON=3.9 per 50 m^2^ (range 0–12), DOT=4.4 per 50 m^2^ (0–12). Second half of summer and early autumn DON=1.8 per 50 m^2^ (0–4), DOT=7.0 per 50 m^2^ (0–21).

Cowdray Estate (Figs. 3G–3L; Table S4): 31 *I. ricinus* ticks were collected (23 along transects), from all life stages. Ticks were collected on five of six transects, on both woodland and downland (Table S4), range 0–7 per 50 m^2^ sampling, DON=6 per 300 m^2^, DOT=8 per 300 m^2^ (Table 4A). One of two downland transects on which a tick was found ran along the South Downs Way. Spring DON=1.7 per 50 m^2^ (range 0–7), DOT=1.8 per 50 m^2^ (0–7). Second half of summer and early autumn DON=0.3 per 50 m^2^ (0–1), DOT=0.3 per 50 m^2^ (0–1).

#### Comparisons between sites

Tick hazard was detected at all four sites surveyed in both 2015 and 2016, but levels (range per sampling, DON, DOT) differed between them, as outlined above and shown in Table 4A. Of the sites at which only *I. ricinus* were collected, The Mens had the highest DON, followed by Queen Elizabeth Country Park, and then Cowdray Estate. The DON of the latter was half that of Queen Elizabeth Country Park, and less than a third of The Mens. These three sites all had greater DON than Seven Sisters Country Park, at which only *H. punctata* were collected. The same ranking of site hazard held for DOT across the four sites. As is evident from Fig. 8, different *I. ricinus* life stages predominate at different seasons, nymphs in spring and larvae in the second half of summer and early autumn. However, the same ranking of site hazard (*i.e*., The Mens > Queen Elizabeth Country Park > Cowdray Estate > Seven Sisters Country Park) also held for DON and DOT from the spring sampling period, and DON and DOT from the sampling period in the second half of summer and early autumn.

Kruskal-Wallis Tests (KWT) showed average ranks for (i) nymphs, and (ii) ticks (all life stages) differed significantly (<0.05) for at least one of the four sites (results, Table 4B). Bonferroni corrected *post-hoc* Dunn’s Tests were used to carry out pairwise site comparisons (Table 4B; sign CIs and magnitudes/directions of differences, Figs. S1 and S2). The Mens had significantly more nymphs than the two other sites at which *I. ricinus* were collected (Cowdray Estate and Queen Elizabeth Country Park), as well as Seven Sisters Country Park at which *H. punctata* was found. In turn, Seven Sisters Country Park had significantly less than Queen Elizabeth Country Park. For ticks (all life stages) The Mens had significantly more ticks than Cowdray Estate, but not the remaining site at which *I. ricinus* was collected (Queen Elizabeth Country Park). However, Queen Elizabeth Country Park and The Mens both had significantly more ticks than Seven Sisters Country Park (where *H. punctata* was collected).

### Habitat associations with tick hazard in the SDNP

Of the four sites drag-sampled in both years, sites with transects entirely in woodland (Queen Elizabeth Country Park, The Mens) had the highest tick hazards (Tables 3 and 4, S2 and S3). However, tick hazard was present at all sites, including on the grazed downland sections of the two sites (Seven Sisters Country Park; Cowdray Estate) which had transects in downland and woodland (Tables S1 and S4). The tick hazard present was not universal in wooded sections of sites; no ticks were found in the forested part of Seven Sisters Country Park (Table S1). A KWT performed of ticks collected (all life stages) on habitat coded transects showed average ranks differed significantly (<0.05) for at least one of the three coded habitat types (results, Table 4C). A Bonferroni corrected *post-hoc* Dunn’s Test (Table 4C; sign CIs and magnitudes/directions of differences, Fig. S3) showed there was not a statistically significant difference in the number of ticks (all life stages) between deciduous woodland and conifer woodland/planting. However, both habitats had significantly more ticks than downland. Similarly, a KWT showed average ranks differed significantly (<0.05) for nymphs (Table 4C), and a Bonferroni corrected *post-hoc* Dunn’s Test (Table 4C; sign CIs and magnitudes/directions of differences, Fig. S4) showed no significant difference between deciduous woodland and conifer woodland/planting. However, as with ticks (all life stages) both habitats had significantly more nymphs than downland.

## Discussion

### Tick hazards, associated pathogens, and general management within the SDNP

*Ixodes ricinus* or *H. punctata* ticks were recorded at all sites drag-sampled, on some transects in high numbers (Figs. 2 and 3). Extent of tick hazard differed between sites. It was significantly higher in woodland compared to grazed downland, but was still present at all downland sites. Our mapping indicates tick hazard is widely distributed across the SDNP, as confirmed by the deerstalker submission of ticks from sites across the National Park.

#### Ixodes ricinus

Given *I. ricinus* (Fig. 5A) has been the tick most often submitted to Public Health England by members of the public or recorded in veterinary surveillance (Jameson & Medlock, 2011; Abdullah et al., 2016; Davies et al., 2017), it is unsurprising it was the species most frequently recovered in the drag-sampling and deerstalker submissions, and the most spatially reported (Fig. 6). The ecology of *I. ricinus* is outlined in our introduction, further information can be found in Földvári (2016) and Kahl & Gray (2023). Based upon the data presented here from the drag-sampling and GIS mapping, it is likely to be responsible for the majority of Lyme disease cases contracted in the SDNP. *Ixodes ricinus* has also been reported to be Europe’s major tick-borne encephalitis vector (Brugger et al., 2017). Tick-borne encephalitis virus has been detected in one of the Park’s host counties, but to our knowledge no ticks within the SDNP have been tested. As included in our Park-wide tick hazard mapping (Fig. 6), Layzell et al. (2018) drag-sampled a single site within the National Park in 2014 (West Dene, West Sussex). They report that they isolated *Borrelia miyamotoi* from *I. ricinus* ticks from that site. Like Lyme disease, *Borrelia miyamotoi* disease is caused by *Borrelia* species, but signs and symptoms markedly differ so that it is classed separately (Telford et al., 2015). *B. miyamotoi* has been detected in *I. ricinus* in countries across Europe (Norway, Finland, Estonia, Latvia, Netherlands, Belgium, France, Germany, Austria, UK, Switzerland, Portugal, Spain, Italy, Serbia, Turkey, Poland, Czech Republic, Slovakia, Hungary, Belarus, Ukraine, Romania, and Russia) with an overall prevalence of 1.0% (95% CI, 1.0–1.1) (Hoornstra et al., 2022). Szekeres et al. (2017) detected it in *I. ricinus* at a site in Germany at a lower prevalence (1.2%) than *B. burgdorferi* s.l. (9.4%) (the cause of Lyme disease), and this pattern of co-residency in *I. ricinus* populations but at lower prevalence has been observed elsewhere (for example by Hansford et al. (2015) in the UK, and Vaculová et al. (2019) in Slovakia). In the USA, a case series of 94 individuals (identified by retrospectively testing stored patient samples) indicated a clinical presentation of chills, headache, generalised/joint pain, thrombocytopenia, and high fever. Of these people, 24% of cases required hospitalisation, and all responded well to antibiotics (Molloy et al., 2015). *Borrelia miyamotoi* disease was discovered far more recently than Lyme disease (the first confirmed Western European case was in 2013 (Fonville et al., 2014)). A clear picture of disease burden in humans is, therefore, not yet available. *Borrelia miyamotoi* detection in the SDNP, in what we found to be the Park’s most well distributed tick vector, adds further weight to the need to conduct interventions.

UK countryside workers perceive spatial overlaps between widening deer abundance and *I. ricinus* (Scharlemann et al., 2008), but wider ecological determinants such as host community compositions affect densities of infected ticks and thus actual disease hazard and determinants vary between site (Kurtenbach et al., 1998; Keesing et al., 2010). For instance, whilst deer likely have roles in most, but not all, UK Lyme disease systems (Ogden, Nuttall & Randolph, 1997; Gilbert et al., 2012) they are non-competent hosts for the pathogen; small mammals/birds are usually required as disease reservoirs (Franke, Hildebrandt & Dorn, 2013). *Ixodes ricinus* is often associated with forests (Ehrmann et al., 2017), and in our fieldwork its presence and densities were highest in wooded areas. However, we also collected the species on sheep-grazed land (as elsewhere in the UK (Evans, Sheals & Macfarlane, 1961; Ogden, Nuttall & Randolph, 1997; Gilbert et al., 2017)). Management strategies across the SDNP should take this into account, especially where downland is bounded by woods (Gilbert et al., 2017). The fieldwork component of our study purposefully focused on county authority managed countryside sites with high numbers of recreational visitors. However, the SDNP also includes within its boundaries some small market towns (*i.e*., Lewes, Midhurst and Petersfield), and studies in both the UK and across Europe have reported presence of *I. ricinus* in urban habitats (*i.e*., parks and gardens) (Rizzoli et al., 2014; Hansford et al., 2022, 2023; Richter et al., 2023). Notably, our SDNP wide mapping did include tick records from gardens. With that in mind, SDNP residents and local authorities in charge of urban green spaces within the Park may benefit from targeted advice on tick awareness and hazard reduction (for an example of the latter aimed partly at residential settings, see Stafford (2004)).

#### Haemaphysalis punctata

*Haemaphysalis punctata’s* (Fig. 5B) continued and expanding presence in the SDNP is evident in our tick hazard mapping (Fig. 6C), raising concerns about pathogens it can vector, including *B. burgdorferi* s.l. (Tälleklint, 1996), tick-borne encephalitis virus (Estrada-Peña & Jongejan, 1999), and spotted fever group rickettsiae. UK *H. punctata* testing has so far been negative for *B. burgdorferi* s.l. (Tijsse-Klasen et al., 2013), to our knowledge unconducted for tick-borne encephalitis virus (known ranges do not presently overlap), but positive for spotted fever group rickettsiae at some sites outside the Park (Tijsse-Klasen et al., 2013). Spotted fever group rickettsiae are an emerging European disease threat (Lindblom et al., 2013), but one that may have been present yet unidentified for some time (Vitale et al., 2006). For example, *Rickettsia massiliae* was first identified as a human pathogen in 2005 after isolation from a clinical sample collected 20 years prior (Vitale et al., 2006). Spotted fever group rickettsiae related misdiagnoses and under-reporting still likely continue (Tijsse-Klasen et al., 2013). *Haemaphysalis punctata* is known to parasitise humans (Hillyard, 1996) and tick submissions to Public Health England by the public show this is happening in the SDNP (Medlock et al., 2018; Phipps, 2019). Sheep and cattle are its main adult hosts, others include horses, hedgehogs, rabbits, birds, goats, deer, and mustelids (Evans, Sheals & Macfarlane, 1961; Hillyard, 1996; Hansford et al., 2019). Despite flock treatments, sheep infestation at Seven Sisters Country Park (one of the sites we collected it at) has been present for decades (personal communication with site sheep farmer, 2015; Medlock et al., 2018). In 2020, 11.5% of a sheep flock in the SDNP near Lewes suffered fatal tick pyaemia, the first such UK outbreak connected to *H. punctata* (Macrelli et al., 2020). Also in 2020, on Brighton’s downland outskirts (near the second site we collected *H. punctata*) sheep used for conservation grazing had to be removed on welfare grounds following heavy infestations with the tick (Phipps et al., 2020). Given these and related incidents Animal Plant Health Agency and Public Health England are investigating further in the Park and working with farmers.

#### Risk of future site co-occupancy by *I. ricinus* and *H. punctata*

Medlock et al. (2018) state that there does not generally seem to be habitat overlap in the UK between *H. punctata* and *I. ricinus*. Though Figs. 6A and 6C show that in all 10 km^2^ OS grid squares where *H. punctata* have been detected *I. ricinus* has also been recorded, this is not in fact in contradiction. On a finer scale of the individual sites JM drag-sampled, tick presence was either *H. punctata* or *I. ricinus*, not both. *I. ricinus* grassland preference in the UK for rough grazing is likely connected to the rapid desiccation it experiences in short grass lacking thick mats of vegetation and litter (Evans, Sheals & Macfarlane, 1961). In contrast, *H. punctata* can likely survive better in short grass, its traditional range includes deserts (Nosek, 1973). Outside the UK *H. punctata* is also found in forest (Borşan et al., 2020). Whilst its established foci in the eastern Downs is relatively unwooded, if allowed to expand its range westward along the downland ridge it will increasingly encounter patchworks of grazing, scrub, and woods. The two species site occupancy and host community may then begin to overlap, with implications for pathogen carry and thus hazard to humans.

#### Comparison with other recreational greenspaces in Southern England

Site tick densities were broadly similar to those measured at some other recreational greenspaces in Southern England. For example, DONs of sites we drag-sampled occupied by *I. ricinus* (respectively 6, 12, and 30 per 300 m^2^, Table 4), were comparable with those reported from drag-sampling in 2018 in Bushy Park (2.31/100 m^2^) and Richmond Park (15.68/100 m^2^) (Hansford et al., 2021). Notably though, the same survey found no ticks at seven other locations (whereas ticks were collected at all our sites). A survey of publicly accessible rural woodland near Bath found a DON of 12.67/100 m^2^ (Hansford et al., 2023), and relatively unusually, also reported DOT: 13.0/100 m^2^ (compared to 8, 22, and 30 per 300 m^2^ at our sites (Table 4)). Dobson, Taylor & Randolph, 2011 sampled recreational sites in the New Forest and Exmoor National Parks. They do not report site DONs, but importantly did find considerable heterogeneity in sampling results at different transects within sites. Van Gestel et al. (2021) found similarly marked within-site differences at recreational sites in Belgium, and so did we (Tables S1–S4). In the next section we outline potential future interventions on a site-by-site basis, but within-site differences in hazard should also be kept in-mind during implementation.

### Recommendations for key locations for future interventions in the SDNP

#### Sites at which *I. ricinus* were collected

Queen Elizabeth Country Park: Given its high tick hazard and very high annual visitor numbers (Table 1) this site is the highest priority for interventions. It also lays in Hampshire where tick-borne encephalitis virus has been detected and would be a logistically simple trial setting for action which may be required elsewhere in the county. Basic measures, such as increased frequency of mowing verges (Medlock et al., 2012; Del Fabbro, 2015) and leaf litter removal (Schulze, Jordan & Hung, 1995) would be implausible across the whole Country Park, but could reduce contact at small high risk plots: *e.g*., edges of marked picnic areas; such as along the path that goes past the visitor centre (Fig. 2G). It hosts large outdoor sports events, and elsewhere tick removal/submission from participants has been used to tick-sample (Hall et al., 2017). Site sampling by this method would be inexpensive, and along with increased signage would raise awareness of tick presence and the value of carrying out post-activity tick-checks. These are important as early tick removal reduces transmission, and during-activity recommendations aimed at individuals to minimise exposure are unlikely to be heeded (Middleton, Cooper & Rott, 2016).

The Mens: The site had the greatest DOT and DON, but could be expected to have the fewest visitors (local conurbations and car park capacity, Table 1). Thus, though its DOT was thirty times Seven Sisters Country Park’s (annual visitors, est. >300,000 (ESCC and SDCB, 2004)), the tick risk to public health is likely to be far smaller. Nevertheless, signage in the carpark would be beneficial and the site could become a useful research/trial location. It’s impressive beech masts (the dominant litter constituent of most of it’s transects) may support high rodent densities, which can be very host-competent for ticks and pathogens, amplifying disease hazard (Keesing et al., 2009; Ostfeld et al., 2014; Krawczyk et al., 2020). Such a relationship has been observed in northeastern USA where high acorn masts cause subsequent year surges in rodents, followed by elevated densities of infected nymphs (DIN) (Ostfeld et al., 2006). Similarly, high beech masting events at a site in Switzerland have been observed to predict increased abundance of *I. ricinus* nymphs 2 years later (Bregnard, Rais & Voordouw, 2020).

Elsewhere, rodent predator protection and reintroduction have been proposed as ecologically beneficial interventions (Nilsen et al., 2007; Levi et al., 2012). For example, Hofmeester et al. (2017) observed an indirect negative correlation of red fox and stone marten activity with DON and DIN at forest plots across the Netherlands, and called for wider predator appreciation and protection. Such an approach may be particularly appropriate to trial at The Mens, given site characteristics. Specifically, (1) its woodland habitat with heavy beech mast, (2) its location within the wider forest matrix of the Weald, and (3) that as a minimal-intervention conservation reserve any action would need to have a presumed low risk of negatively affecting ecosystem health (unlike for example, acaracide spraying (Middleton, Cooper & Rott, 2016)). Tick pathogen testing could establish if the very high densities of ticks at The Mens are matched by high densities of infected ticks. If so, measurement of vertebrate, especially rodent, tick and pathogen burden would determine if predator re-introduction (*i.e*., pine martens) and protection (*i.e*., foxes) should be trialled as health interventions at the site and environs. As regards pine martens, a small breeding population was confirmed in the New Forest National Park in 2022 (HIWWT, 2022), but the species is otherwise absent from South-East England. However, recovery efforts are being supported by wildlife bodies (MacPherson & Wright, 2021), and there is growing interest locally (including from the landowner of The Mens (SWT, 2023)). If a trial is successful in reducing tick-borne disease hazard, it could be extended further through the wooded wealden section of the SDNP of which The Mens is a part. This bioregion has been identified as having high habitat suitability for pine marten reintroduction, unlike most of the downland section of the Park (see maps in: MacPherson & Wright, 2021). In contrast to pine martens, the whole SDNP is suitable for fox populations. However, against a backdrop of seeming national decline (Sainsbury et al., 2019), they continue to be culled by farmers and others across the Park.

Given The Mens is a relatively large biodiverse reserve for South-East England (Whitbread, 2013), if infection densities are lower than expected based on tick densities, explanatory work could contribute to scientific debate on relationships between biodiversity and health (see: Randolph & Dobson, 2012; Levy, 2013; Foley & Piovia-Scott, 2014; Civitello et al., 2015; Ruyts et al., 2018; Wang et al., 2023).

Cowdray Estate and Deerstalkers: With comparably low tick hazard and likely fewer visitors than most other sites (Table 1), the Estate itself does not need to carry out interventions to reduce site tick hazard. However, action aimed at walkers crossing Cowdray Estate and deerstalkers working in it (and by implication elsewhere under similar circumstances) is warranted. Publicly maintained car parking along the route of the South Downs Way in Cowdray Estate would benefit from tick related signage (absent during our visits). To our knowledge no study of *B. burgdorferi* s.l. seropositivity or Lyme disease cases has been conducted for professional UK deerstalkers as has been for more commonly considered occupationally at risk groups such as foresters (*e.g*., De Keukeleire et al., 2018). However, British Deer Society volunteers at multiple sites stated to the lead author of this article that during stalking and butchery they regularly encounter ticks, often getting bitten. This statement is plausible given data presented in Table 2 and Fig. 7. On a precautionary principle, professional deerstalkers would thus benefit from provision of permethrin-impregnated clothing which is effective against ticks (Faulde et al., 2015), and targeted tick-related education. They would also be a priority group for future tick-related disease vaccination efforts. A vaccine against European *B. burgdorferi* s.l. strains is under development (Nayak et al., 2020), and one for tick-borne encephalitis is available but only recommended at present for those doing outdoor activities in a country where tick-borne encephalitis virus is common (NHS, 2021).

#### Sites at which *H. punctata* were collected

Seven Sisters Country Park and Ditchling Beacon Nature Reserve*: Haemaphysalis punctata’s* original distribution at Cuckmere Haven and surroundings suggests importation *via* migratory birds (Tijsse-Klasen et al., 2013), but how it is spreading westward is unclear. This may be facilitated by livestock movements (including conservation grazing (Medlock et al., 2018)), birds (immature tick stages), or pets (Public Health England has received submissions taken off dogs locally (Phipps, 2019)). Public Health England and Animal Plant Health Authority are carrying out targeted surveys to determine its invasion boundary (Fig. 6C) and means of spread, knowledge required for effective region-level intervention. Additional work to understand its ecology and control is needed (Medlock et al., 2018). Acaricide application to sheep on the Lewes site resolved the situation in-year (Phipps et al., 2020). However, our data showing continued *H. punctata* presence at Seven Sisters Country Park despite reported flock acaricide application (see above) suggests livestock treatment alone may sometimes be insufficient. This could potentially be because *H*. *punctata* instars are supported by non-domestic hosts. Pasture spelling (*i.e*., temporary flock removal for *c*. 6 months) is a traditional practice to reduce tick numbers (Hillyard, 1996), yet may be unsuccessful in clearing *H*. *punctata* from grazing land given unfed individuals can survive relatively long periods without blood meals: nymphs, 252 days; adults, 255 (Evans, Sheals & Macfarlane, 1961). Trials at Seven Sisters Country Park, Ditchling Beacon Nature Reserve, and related sites could evaluate approaches for control whilst reducing on-site tick hazards. No tick-related notices were seen during sampling or subsequent visits (up to January 2021) at either site. Instructive, but not alarming, signs could be placed in carparks at both, as has been done unobtrusively at another site, Mount Caburn, which is a less visited local site, but where *H*. *punctata* is also present. Notices should emphasise strongly the need for post visit checks, including vigilance over longer subsequent periods than normally recommended (*H. punctata* nymphs usually feed for 1 week, but may attach up to 33 days (Evans, Sheals & Macfarlane, 1961)).

### Recommendations for actions beyond the SDNP

This work is part of a regional intervention planning exercise, so findings are highly site specific limiting generalisation. However, this study may be useful as a model for intervention planning elsewhere, and the data is available for meta-analyses. Specifically, we would encourage National Park authorities in the UK and beyond to directly support similar surveillance and planning exercises in their territories as a foundation for taking action when tick hazard is present. We are cautious about making recommendations for specific control measures beyond the bioregions our study is grounded in. Nevertheless, some of the management suggestions we have provided may be appropriate elsewhere. For example, edge-mowing alongside the most heavily used paths in country parks, and protective acaracide-treated clothing for countryside workers. These have minimal cost implications and potentially high impacts in reducing exposure (de Groot, 2016; Verheyen & Ruyts, 2016), so adoption would seem reasonable based on existing (though limited) evidence. In contrast, the larger more landscape-scale interventions we have recommended for some of our drag-sampled sites (*i.e*., predator introduction; pasture spelling) could be expected to have more implications, and whilst there is good reason to think they may be effective at some sites (with some tick species) (Hillyard, 1996; Hofmeester et al., 2017), the evidence base is not yet strong. We would still encourage others to consider these measures if they are managing similar habitats to the SDNP (deciduous woodland with high masting, sheep grazed grasslands, *etc*.), but only as part of rigorous trials. This is our intention in the South Downs, and we are keen to be in contact with others doing similar projects so that we can build the evidence base together.

### Strengths and weaknesses

One study strength was deerstalker involvement, whose submissions enabled responsive site selection and contributed to SDNP wide tick hazard mapping. Another strength was our use of density of ticks, rather than only density of nymphs. Starting in the USA with Lyme disease the latter is more common (Han et al., 2021). However, *H. punctata* presence in the Park illustrates the reduced appropriateness of reporting only density of nymphs for European work. Transovarial transmission of spotted fever group rickettsiae is established, Macaluso & Paddock (2014) so a hazard metric that excludes larvae (as density of nymphs does) will under-count vector density for spotted fever group rickettsiae and other pathogens (*e.g*., *B. miyamotoi* (Hansford et al., 2015)). Similarly, tick-borne encephalitis virus cycles require larvae-nymph co-feeding (PHE, 2019). The idea, based on US data, that transovarial transmission of *B. burgdorferi* s.l. is rare or non-existent (Ostfeld, 2011, p.43) is the main basis on which density of nymphs has been used for Lyme disease site hazard assessment. However, Van Duijvendijk et al. (2016) showed *Borrelia afzelii, a* common cause of Lyme disease in Europe which is not present in the US, can be transmitted by *I. ricinus* larvae. In a UK study by Hall et al. (2017), 0.7% were *B. burgdorferi* s.l. positive. A study weakness concerns number and months of repeat sampling. Firstly, our first four sites were intended to be drag-sampled twice per year, for 2 years. However, this was not possible for one site, which was sampled three times only, and with one sampling-day delayed (Fig. 8). Secondly, some species lifecycles (most notably, *I. ricinus*) cause seasonal differences in questing tick numbers/life-stage proportions. However, for logistical reasons the months’ individual site samplings took place in varied. This was mainly due to between-site distances and the need to avoid sampling during or after rain (which reduces tick questing and drag-sampling efficacy), which in both seasonal sampling periods is generally highly frequent and changeable spatially across the 140 km length of the Park. This reduces confidence in validity of site comparisons somewhat, though this is partially balanced by in-year and multiple-year repeat samplings. Site DON and DOT measures from both seasonal sampling periods accorded with rankings of overall DON and DOT per site, suggesting multi-year rankings were not overly skewed by sampling-date difference. Nevertheless, increased confidence in inter-site comparisons could be obtained in future studies by simultaneous sampling by multiple fieldworkers working across the sites on days suitable for cross-Park sampling. In this study, intervals between site samplings were not uniform, which introduces a risk that a depletion effect may differentially bias measures of hazard across sites. Future work would thus benefit from fixed uniform intervals, which could be enabled by fieldworkers working simultaneously across the Park.

We did not ourselves survey visitor numbers, and such data was not available from land managers for most sites. For this reason, though we do infer which sites are the priority for public health measures based partly on visitor related data (annual visitor totals, when available; relative site difference in carpark capacity; nearby conurbations), we could not quantify tick risk itself. To enable this, similar studies would benefit from parallel site surveys of tick densities (*i.e*., hazards) and visitor numbers (*i.e*., exposures), though this would require increased resource. GIS tick hazard mapping successfully brought together all publicly available, and some unpublished, tick records from SDNP. However, to our knowledge vouchers are unavailable for the historic and pan-species records included, leaving some uncertainty regarding correct species identification. In contrast a strength of our fieldwork is our vouchers.

## Conclusions

We set out to map tick hazard distribution across the SDNP, analyse habitat associations, identify and describe potential key locations for future interventions and determine their tick hazard (species and density). Against a background of increased concern about tick-borne pathogens in the UK (*B. burgdorferi* s.l., *B. miyamotoi*, *B. venatorum*, tick-borne encephalitis virus, spotted fever group rickettsiae), our mapping shows tick hazard is broadly distributed across SDNP. *Ixodes ricinus* was the most common tick found, though the potential range expansion of *H. punctata* from its historic foci at SDNPs far east is concerning, not least as it seems better able to thrive on grazed downland than *I. ricinus*. Our study confirms woodland is the habitat in the SDNP most associated with tick hazard, but ticks (including *I. ricinus*) were collected on downland, and if *H. punctata* is allowed to expand its range westward, this is only likely to increase. Tick hazard does not reflect negatively on land managers, but should be a stimulus for action, especially at those sites with high tick risk: *i.e*., those with high DOT/DON and high visitor numbers. We identified key potential sites for interventions and based on measured tick-density, site description, and visitor data have provided site specific recommendations for control measures (which should be evaluated *in-situ* during roll-out) and future research. These include targeted management at small high tick hazard plots with expected heavy visitor numbers (Queen Elizabeth Country Park), signage to increase awareness of post-visit precautions (all sites), repellent impregnated clothing for deerstalkers (Cowdray Estate; wherever else in the Park deerstalkers are active), and flock-based experimental trials to control *H. punctata* (Seven Sisters Country Park, Ditchling Beacon Nature Reserve). Further research at one of the sites with very high tick density (The Mens) may valuably contribute to an understanding of ecological dynamics underlying infection density, and potential use of predator re-introduction and protection as a public health intervention. Ecological research on *H. punctata* in the UK (including determination of seasonal life-stage distribution) would contribute towards control strategies. Whilst interventions are necessarily site-specific, this does create the danger of implementation becoming fragmented. However, SDNPA is ideally placed to link and champion site-based and regional policies to reduce hazard, whilst avoiding or reducing conflict between public health and ecosystem health.

## Supplemental Information

10.7717/peerj.17483/supp-1 Supplemental Information 1 Safety measures, GIS layer generation, carparks at drag-sampled sites, and justification of analysis.

10.7717/peerj.17483/supp-2 Supplemental Information 2 Ticks collected at Seven Sisters Country Park, 2015 and 2016. Ticks were collected through combined sampling with woollen blanket (B), chap (C), and flags (F). NC=not collected.

10.7717/peerj.17483/supp-3 Supplemental Information 3 Ticks collected at Queen Elizabeth Country Park, 2015 and 2016. Ticks were collected through combined sampling with woollen blanket (B), chap (C), and flags (F). *2 removed for safety validation before light microscopy confirmation of ID. NC=not collected. SDW = South Downs Way national trail.

10.7717/peerj.17483/supp-4 Supplemental Information 4 Ticks collected at The Mens, 2015 and 2016. Ticks were collected through combined sampling with woollen blanket (B), chap (C), and flags (F). *2 removed for safety validation before light microscopy confirmation of ID. NC=not collected.

10.7717/peerj.17483/supp-5 Supplemental Information 5 Ticks collected at Cowdray Estate, 2015 and 2016. Ticks were collected through combined sampling with woollen blanket (B), chap (C), and flags (F). *1 removed for safety validation before light microscopy confirmation of ID. NC=not collected. SDW = South Downs Way national trail.

10.7717/peerj.17483/supp-6 Supplemental Information 6 Sign CIs and magnitudes/directions of differences from Bonferroni corrected *post-hoc* Dunn’s Tests on tick (all life stages) hazard at four sites sampled both 2015 and 2016. N=90. 1=The Mens; 2=Cowdray Estate; 3=Seven Sisters Country Park; 4=Queen Elizabeth Country Park.

10.7717/peerj.17483/supp-7 Supplemental Information 7 Sign CIs and magnitudes/directions of differences from Bonferroni corrected *post-hoc* Dunn’s Tests on nymph hazard at four sites sampled both 2015 and 2016. 1=The Mens; 2=Cowdray Estate; 3=Seven Sisters Country Park; 4=Queen Elizabeth Country Park.

10.7717/peerj.17483/supp-8 Supplemental Information 8 Sign CIs and magnitudes/directions of differences from Bonferroni corrected *post-hoc* Dunn’s Tests on ticks (all life stages) collected on transects with differing habitats at the four sites sampled 2015 and 2016. N=90. 1=deciduous woodland; 2=Conifer woodland/planting; 3=Sheep grazed downland.

10.7717/peerj.17483/supp-9 Supplemental Information 9 Sign CIs and magnitudes/directions of differences from Bonferroni corrected *post-hoc* Dunn’s Tests on nymphs collected on transects with differing habitats at the four sites sampled 2015 and 2016. N=90. 1=deciduous woodland; 2=Conifer woodland/planting; 3=Sheep grazed downland.

## Additional Information and Declarations

### Field Study Permissions

The following information was supplied relating to field study approvals (*i.e*., approving body and any reference numbers): Ticks were collected at field sites with the permission of the landowners. These were: East Sussex County Council, Hampshire County Council, Sussex Wildlife Trust, and Cowdray Estate.

### Data Availability

The following information was supplied regarding data availability: The full tick data for each of the drag sampled sites is available in the Supplemental Files. The study data are available at OSF: Middleton, Jo. 2024. “Tick Hazard in the South Downs National Park (UK): Species, Distribution, Key Locations for Future Interventions, Site Density, Habitats.” OSF. May 9. osf.io/vxyqc.
