# Supplementary material for: Tick hazard in the South Downs National Park (UK): species, distribution, key locations for future interventions, site density, habitats
Source: PeerJ. 2024 Jun 12;12:e17483. doi: 10.7717/peerj.17483 (PMC11179636; doi:10.7717/peerj.17483)
Supplement: Supplemental Information 1 [file peerj-12-17483-s001.docx]

**Supplementary Article**: Middleton, Cooper, Rott. 2024. Tick hazard in the South Downs National Park (UK): species, distribution, key locations for future interventions, site density, habitats. *PeerJ*.

**Safety measures**

*Researcher safety and cross-contamination control*

So JM could better see and remove any ticks that attached to them during drag-sampling, white collection clothes and wellington boots (taped to trousers) were worn. These were removed on-site, and sealed in a container with the collection materials. Clothing and collection blankets, chaps, and flags were machine washed at high heat and dried between uses, which reliably kills ticks (Nelson *et al.*, 2016). These measures also reduced the risk of cross-contaminating sites with tick or pathogen species, a potential hazard parallel to concerns raised by Dunn (2014) about invasive aquatic species and inadequate biosecurity in ecological fieldwork.

JM was not aware of having been bitten during the study, or developing any signs or symptoms indicating borreliosis. Blood samples were taken at JM’s general practice before field season one, between field seasons, and after field season two. LD seropositivity was tested for at Brighton and Sussex University Hospitals NHS Trust. All tests were negative, suggesting JM had not contracted borreliosis during the study.

Where transects chosen through randomisation would have been dangerous to drag-sample randomisation was repeated until OS Grid references for safe transects arose. This was required once at Ditchling Beacon (very steep slope over a road) and twice at Seven Sisters Country Park (in the waters of Cuckmere).

Dunn A. 2014. Invasive species and invasive diseases: parallels and interactions. Disease Ecology: British Society of Parasitology Autumn Symposium 2014, 18 September 2014 University of Salford.

Nelson CA, Hayes CM, Markowitz MA, Flynn JJ, Graham AC, Delorey MJ, Mead PS, Dolan MC. 2016. The heat is on: Killing blacklegged ticks in residential washers and dryers to prevent tickborne diseases. *Ticks and Tick-borne Diseases* 7:958. DOI: 10.1016/j.ttbdis.2016.04.016.

*Deerstalker submissions*

Deerstalkers were asked to put on disposable gloves, inspect the whole animal (stating ‘part exam’ on the form if that was not possible), collect every visible tick (using the supplied ‘tick twister’), and place them in pre-coded 1ml cryovials, pre-filled with 70% ethanol, before disposing of gloves and washing hands/using supplied alcohol hand gel. Deerstalkers were given information sheets from Public Health England on signs and symptoms to look out for and when to seek general practitioner advice.

Cryovials containing ticks were returned by post in pre-prepared, pre-paid envelopes within sealed bags, wrapped in bubble wrap and absorbent secondary packaging (in line with Royal Mail requirements).

**GIS layer generation**

In addition to individual ticks submitted by the public, Public Health England/Health Protection Agency maps also include (marked as such) the historic biodiversity records now also available in National Biodiversity Network Atlas. However, Public Health England/Health Protection Agency maps are at 10 km^2^ resolution, which does not allow confirmation records are from within the South Downs National Park for those 10 km grid squares at its border which include land outside as well as in the Park. Given this, historic data was taken from NBN Atlas which has point records. Where historic point records were outside the Park, grid squares involved were not marked positively for recorded presence. The same approach was taken to point data extracted from Medlock *et al*. (2018). Those individual OS 10 km grid squares with presence records from pre-2005 only were highlighted on our maps with latest record dates.

**Carparks at drag-sampled sites**

JM measured all 15 carparks on 25^th^ December 2023, when they could be expected to be largely empty. No changes to the carparks had been made in the period between the tick survey and carpark measurements. As only Queen Elizabeth Country Park had individual marked-out car spaces in its carparks, space per m^2^ ratios from it were used to estimate capacity of each of the other sites. In each case, the ratio chosen was from the Queen Elizabeth Country Park carpark closest in square metres to the unmarked site carpark.

| **Site** | **Carpark** | **Square metres** | **Carpark spaces** |  |
| --- | --- | --- | --- | --- |
|  |  |  | *Counted* | *Space to m^2^ ratio* |
| **Queen Elizabeth Country Park (QECP)** | Juniper (J) | 2400 | 107 | 1 to 22.4 |
|  | Lower Juniper (LJ) | 404 | 13 | 1 to 31.1 |
|  | Upper Windmill (UW) | 1208 | 42 | 1 to 28.8 |
|  | Lower Windmill (LW) | 1134 | 35 | 1 to 32.4 |
|  | Benham Bushes (BB) | 1110 | 42 | 1 to 26.4 |
|  | Gravel Hill (GH) | 750 | 32 | 1 to 23.4 |
|  | Lower Main (LM) | 1411 | 57 | 1 to 24.8 |
|  | Main (M) | 1530 | 73 | 1 to 21.3 |
|  | **Totals** | **9947** | **401** | **1 to 24.8** |
|  |  |  | *Estimated* | *QECP ratio used* |
| **Seven Sisters Country Park** | River | 1820 | 85 | M |
|  | Forest | 4437 | 198 | J |
|  | Canoe jetty | 285 | 9 | LJ |
|  | **Totals** | **6542** | **292** |  |
| **Ditchling Beacon Nature Reserve** | Upper | 806 | 34 | GH |
|  | Lower | 301 | 10 | LJ |
|  | **Totals** | **1107** | **44** |  |
| **Cowdray Estate** | Cocking, South Downs Way | 315 | **10** | LJ |
| **The Mens** | Sussex Wildlife Trust | 175 | **6** | LJ |

**Justification of analysis**

Non-parametric testing was carried out to determine the significance of any difference between tick hazards (1) at the four sites, and (2) at transects with differing coded habitat types. Non-parametric testing was used for two reasons. Firstly, the number of samples covering the four sites (90 samplings, 24 transects) may not have adequately captured a normal population distribution, if one was present. Secondly, given the study concerned the collection of parasitic organisms (often grouped) whose exact siting is highly dependent on placement by sparse hosts, data would not be expected to be normally distributed. Ditchling Beacon Nature Reserve, sampled only in 2016, was not included in statistical analysis as conclusions based on single year tick samplings have limited validity (Dobson*,* Taylor & Randolph, 2011).
