# Supplementary material for: Tick hazard in the South Downs National Park (UK): species, distribution, key locations for future interventions, site density, habitats
Source: PeerJ. 2024 Jun 12;12:e17483. doi: 10.7717/peerj.17483 (PMC11179636; doi:10.7717/peerj.17483)
Supplement: Supplemental Information 2 — Ticks were collected through combined sampling with woollen blanket (B), chap (C), and flags (F). NC=not collected. [file peerj-12-17483-s002.docx]

| Plot  and  Habitat | Dominant vegetation; under-growth;  main litter | Date | Undergrowth (height cm) | Rh % | | T ^O^C | | Ticks Collected  (*H. punctata*) | | | |
| --- | --- | --- | --- | --- | --- | --- | --- | --- | --- | --- | --- |
|  |  |  |  | **50cm** | **Litter** | **50cm** | **Litter** | **Larvae** | **Nymphs** | **Adults** | **Totals** |
| *Fig.* 2A  Downland  (sheep grazed, busy footpath) | Grass; dense grass; no visible litter | 5.6.15 | 70 | 69 | 74 | 20 | 26 |  |  |  | 0 |
|  |  | 29.9.15 | 10 | 57 | 90 | 17 | 20 |  |  |  | 0 |
|  |  | 2015 transect totals | | | | | |  |  |  | 0 |
|  |  | 30.4.16 | 5 | 60 | 74 | 11 | 11 |  |  |  | 0 |
|  |  | 24.8.16 | NC | 43 | 50 | 29 | 28 |  |  |  | 0 |
|  |  | 2016 transect totals | | | | | |  |  |  | 0 |
|  |  | **Transect totals (range)** | | | | | |  |  |  | **0 (0-0)** |
| *Fig*. 2B  Downland  (sheep grazed) | Grass; dense grasses, nettles; no visible litter | 5.6.15 | 0 | 75 | 80 | 19 | 19 |  |  |  | 0 |
|  |  | 29.9.15 | 3-5 | 57 | 67 | 18 | 19 |  |  |  | 0 |
|  |  | 2015 transect totals | | | | | |  |  |  | 0 |
|  |  | 30.4.16 | 2-15 | 62 | 65 | 10 | 12 |  |  |  | 0 |
|  |  | 24.8.16 | NC | 42 | 62 | 30 | 31 |  |  |  | 0 |
|  |  | 2016 transect totals | | | | | |  |  |  | 0 |
|  |  | **Transect totals (range)** | | | | | |  |  |  | **0 (0-0)** |
| *Fig*. 2C  Downland  (sheep grazed) | Grass; dense grass; no visible litter | 5.6.15 | 0 | 66 | 76 | 17 | 19 |  | 2B |  | 2B |
|  |  | 29.9.15 | 15-30 | 57 | 66 | 17 | 16 |  |  |  | 0 |
|  |  | 2015 transect totals | | | | | |  | 2 |  | 2 |
|  |  | 30.4.16 | 5 | 52 | 83 | 11 | 11 |  |  |  | 0 |
|  |  | 24.8.16 | 35 | 45 | 50 | 29 | 31 |  |  |  | 0 |
|  |  | 2016 transect totals | | | | | |  |  |  | 0 |
|  |  | **Transect totals (range)** | | | | | |  | **2** |  | **2 (0-2)** |
| *Fig*. 2D  Woodland  (High canopy, carpark picnic area) | Beech, conifer; no undergrowth; conifer and beach litter | 10.6.15 | 0 | 50 | 52 | 17 | 17 |  |  |  | 0 |
|  |  | 29.9.15 | 0 | 57 | 75 | 17 | 16 |  |  |  | 0 |
|  |  | 2015 transect totals | | | | | |  |  |  | 0 |
|  |  | 30.4.16 | 0 | 49 | 83 | 12 | 12 |  |  |  | 0 |
|  |  | 24.8.16 | 0 | 49 | 56 | 26 | 26 |  |  |  | 0 |
|  |  | 2016 transect totals | | | | | |  |  |  | 0 |
|  |  | **Transect totals (range)** | | | | | |  |  |  | **0 (0-0)** |
| *Fig*. 2E  Woodland  (multi-level canopy, next to visitor carpark) | Sycamore; sycamore saplings, nettles, ivy; sycamore leaves | 10.6.15 | 100 | 46 | 50 | 18 | 18 |  |  |  | 0 |
|  |  | 29.9.15 | 100 | 62 | 80 | 17 | 16 |  |  |  | 0 |
|  |  | 2015 transect totals | | | | | |  |  |  | 0 |
|  |  | 30.4.16 | 15-100 | 50 | 69 | 12 | 14 |  |  |  | 0 |
|  |  | 24.8.16 | 100 | 52 | 56 | 27 | 25 |  |  |  | 0 |
|  |  | 2016 transect totals | | | | | |  |  |  | 0 |
|  |  | **Transect totals (range)** | | | | | |  |  |  | **0 (0-0)** |
| *Fig*. 2F  Downland  (sheep grazed, busy path) | Grass; thistles, dense grasses; no visible litter | 5.6.15 | 50 | 56 | 67 | 18 | 20 |  |  | 2♀C | 2C |
|  |  | 29.9.15 | 50 | 59 | 87 | 19 | 20 |  |  |  | 0 |
|  |  | 2015 transect totals | | | | | |  |  | 2 | 2 |
|  |  | 30.4.16 | 5 | 44 | 65 | 12 | 14 |  |  |  | 0 |
|  |  | 24.8.16 | 6 | 35 | 42 | 31 | 32 |  |  |  | 0 |
|  |  | 2016 transect totals | | | | | |  |  |  | 0 |
|  |  | **Transect totals (range)** | | | | | |  |  | **2** | **2(0-2)** |
| Site transect totals  (mean, range, IQR) | | | | | | | |  | **2** | **2♀** | **4 (1,**  **0-2, 0-2)** |
| Extras | | 2015 (all B) | | | | | |  | 6 | 1♀ | 7 |
|  |  | 2016 (all B) | | | | | | 65 | 2 |  | 67 |
|  |  | **Total extra ticks collected** | | | | | | **65** | **8** | **1♀** | **74** |
| Total ticks collected at site | | | | | | | | **65** | **10** | **3♀** | **78** |
