# Supplementary material for: Tick hazard in the South Downs National Park (UK): species, distribution, key locations for future interventions, site density, habitats
Source: PeerJ. 2024 Jun 12;12:e17483. doi: 10.7717/peerj.17483 (PMC11179636; doi:10.7717/peerj.17483)
Supplement: Supplemental Information 3 — Ticks were collected through combined sampling with woollen blanket (B), chap (C), and flags (F). *2 removed for safety validation before light microscopy confirmation of ID. NC=not collected. SDW = South Downs Way national trail. [file peerj-12-17483-s003.docx]

| Plot  and  Habitat | Dominant vegetation; under-growth;  main litter | Date | Undergrowth (height cm) | Rh % | | T ^O^C | | Ticks Collected  (*I. ricinus*) | | | |
| --- | --- | --- | --- | --- | --- | --- | --- | --- | --- | --- | --- |
|  |  |  |  | **50cm** | **Litter** | **50cm** | **Litter** | **Larvae** | **Nymphs** | **Adults** | **Totals** |
| *Fig*. 2G  Woodland  (high canopy, SDW from visitor cnt.) | Beech, ash; nettles; beech litter | 2.8.15 | 40 | 39 | 44 | 24 | 24 |  | 1B |  | 1 |
|  |  | 6.9.15 | 80 | 54 | 69 | 14 | 15 |  | 1B | 1♀B | 2B |
|  |  | 2015 transect totals | | | | | |  | 2 | 1 | 3 |
|  |  | 24.4.16 | nc | 56 | 82 | 9 | 9 |  | 2B |  | 2B |
|  |  | 24.9.16 | nc | 67 | 83 | 18 | 17 |  | 3B |  | 3B |
|  |  | 2016 transect totals | | | | | |  | 5 |  | 5 |
|  |  | **Transect totals (range)** | | | | | |  | **7** | **1** | **8 (0-3)** |
| *Fig.* 2H  Woodland  (high canopy) | Conifer, beech, yew; nettle patches; conifer & beach litter | 2.8.15 | 0 | 44 | 55 | 22 | 22 |  |  |  | 0 |
|  |  | 6.9.15 | 0-80 | 59 | 76 | 15 | NC | 2B | 1B |  | 3B |
|  |  | 2015 transect totals | | | | | | 2 | 1 |  | 3 |
|  |  | 24.4.16 | 0-50 | 49 | 81 | 10 | 9 |  |  |  | 0 |
|  |  | 24.9.16 | 0-100 | 66 | 99 | 18 | 16 | 10B | 3B |  | 13B |
|  |  | 2016 transect totals | | | | | | 10 | 3 |  | 13 |
|  |  | **Transect totals (range)** | | | | | | **12** | **4** |  | **16 (0-13)** |
| *Fig*. 2I  Woodland  (high canopy) | Beech, yew, conifer; brambles, grass; beech and conifer litter | 15.6.15 | 0-20 | 61 | 69 | 17 | 16 |  | 1B, 1C |  | 1B, 1C |
|  |  | 2.8.15 | 60 | 50 | 71 | 22 | 24 |  | 1B, 1C | 1♂B | 2B, 1C |
|  |  | 2015 transect totals | | | | | |  | 4 | 1 | 5 |
|  |  | 24.4.16 | 60 | 45 | 83 | 9 | 11 |  | 8B, 2C | 1♂B | 9B, 2C |
|  |  | 24.9.16 | nc | 68 | 88 | 19 | 17 | 24B | 1B |  | 25B |
|  |  | 2016 transect totals | | | | | | 24 | 11 | 1 | 36 |
|  |  | **Transect totals (range)** | | | | | | **24** | **15** | **2** | **41 (2-25)** |
| *Fig.* 2J  Woodland  (high canopy, 20m from picnic area) | Beech; beech saplings, nettles; beach mast and leaves, | 15.6.15 | 0-100 | 57 | 66 | 18 | 17 |  | 1B |  | 1B |
|  |  | 2.8.15 | 30 | 46 | 72 | 20 | 20 |  |  |  | 0 |
|  |  | 2015 transect totals | | | | | |  | 1 |  | 1 |
|  |  | 24.4.16 | 0 | 39 | 52 | 11 | 12 |  |  |  | 0 |
|  |  | 24.9.16 | 0 | 60 | 84 | 19 | 17 |  |  |  | 0 |
|  |  | 2016 transect totals | | | | | |  |  |  | 0 |
|  |  | **Transect totals (range)** | | | | | |  | **1** |  | **1 (0-1)** |
| *Fig.* 2K  Woodland  (footpath from car park) | Grass and nettles; | 15.6.15 | 70 | 66 | 72 | 18 | 17 |  |  |  | 0 |
|  |  | 2.8.15 | 100 | 43 | 53 | 21 | 21 |  |  |  | 0 |
|  |  | 2015 transect totals | | | | | |  |  |  | 0 |
|  |  | 24.4.16 | 21 | 45 | 86 | 10 | 11 |  |  |  | 0 |
|  |  | 24.9.16 | 10-75 | 71 | 86 | 18 | 18 |  |  |  | 0 |
|  |  | 2016 transect totals | | | | | |  |  |  | 0 |
|  |  | **Transect totals (range)** | | | | | |  |  |  | **0 (0-0)** |
| *Fig*. 2L  Woodland  (low canopy) | Beech, hazel, yew; brambles, beech saplings; beech litter | 2.8.15 | 20 | 54 | 60 | 20 | 20 |  | 5B |  | 5B |
|  |  | 6.9.15 | 20 | 63 | 73 | 14 | 14 |  |  |  | 0 |
|  |  | 2015 transect totals | | | | | |  | 5 |  | 5 |
|  |  | 24.4.16 | 60 | 59 | 90 | 9 | 9 |  | 10B, 4C | 1♂B | 11B, 4C |
|  |  | 24.9.16 | nc | 79 | 88 | 17 | 16 | 1B | 1B | 1♂B | 3B |
|  |  | 2016 transect totals | | | | | | 1 | 15 | 2 | 18 |
|  |  | **Transect totals (range)** | | | | | | **1** | **20** | **2** | **23 (1-15)** |
| Site transect totals  (mean, range, IQR) | | | | | | | | **37** | **47** | **1♀, 4♂** | **89 (15,**  **0-41,1-28)** |
| Extras | | 2015 (all B) | | | | | | 16 | 45* | 2♀ | 63 |
|  |  | 2016 (all B) | | | | | | 9 | 20* | 1♀, 1♂ | 31 |
|  |  | **Total extra ticks collected** | | | | | | **25** | **65** | **3♀, 1♂** | **94** |
| Total ticks collected at site | | | | | | | | **62** | **112** | **4♀, 5♂** | **183** |
