# Supplementary material for: Tick hazard in the South Downs National Park (UK): species, distribution, key locations for future interventions, site density, habitats
Source: PeerJ. 2024 Jun 12;12:e17483. doi: 10.7717/peerj.17483 (PMC11179636; doi:10.7717/peerj.17483)
Supplement: Supplemental Information 4 — Ticks were collected through combined sampling with woollen blanket (B), chap (C), and flags (F). *2 removed for safety validation before light microscopy confirmation of ID. NC=not collected. [file peerj-12-17483-s004.docx]

| Plot  and  Habitat | Dominant vegetation; under-growth;  main litter | Date | Undergrowth (height cm) | Rh % | | T ^O^C | | Ticks Collected  (*I. ricinus*) | | | |
| --- | --- | --- | --- | --- | --- | --- | --- | --- | --- | --- | --- |
|  |  |  |  | **50cm** | **Litter** | **50cm** | **Litter** | **Larvae** | **Nymphs** | **Adults** | **Totals** |
| *Fig*. 3A  Woodland  (footpath from car park with grass verge) | Beech; grass verge on path; beech mast and leaves | 10.5.15 | 30 | 67 | 72 | 17 | 16 |  | 10B | 2♂B | 12B |
|  |  | 19.6.15 | 0 | 59 | 63 | 16 | 16 |  | 2B |  | 2B |
|  |  | 2015 transect totals | | | | | |  | 12 | 2 | 14B |
|  |  | 23.4.16 | 5-37 | 54 | 74 | 10 | 10 |  | 12B |  | 12B |
|  |  | 30.8.16 | 15 | 66 | 88 | 19 | 18 | 1B | 1B |  | 2B |
|  |  | 2016 transect totals | | | | | | 1 | 13 |  | 14B |
|  |  | **Transect totals (range)** | | | | | | **1** | **25** | **2** | **28 (2-12)** |
| *Fig.* 3B  Woodland  (high canopy) | Beech; sparse holly in vicinity, not on plot;  beech mast and leaves, holly leaves | 10.5.15 | 0 | 68 | 76 | 16 | 17 |  | 3B |  | 3B |
|  |  | 19.6.15 | 0 | 59 | 63 | 16 | 16 |  | 1B |  | 1B |
|  |  | 2015 transect totals | | | | | |  | 4 |  | 4 |
|  |  | 23.4.16 | 0 | 41 | 84 | 10 | 11 |  |  |  | 0 |
|  |  | 30.8.16 | 0 | 65 | 84 | 20 | 19 | 4B | 2B |  | 6B |
|  |  | 2016 transect totals | | | | | | 4 | 2 |  | 6 |
|  |  | **Transect totals (range)** | | | | | | **4** | **6** |  | **10 (0-6)** |
| *Fig*. 3C  Woodland  (multi-level canopy) | Beech, oak; holly samplings; beach and oak mast and leaves, holly leaves | 10.5.15 | 50-60 | 60 | 76 | 17 | 17 |  | 1C,7B | 1♂B | 8B, 1C |
|  |  | 19.6.15 | nc | 58 | 61 | 17 | 17 |  | 1C,1B |  | 1B, 1C |
|  |  | 2015 transect totals | | | | | |  | 10 | 1 | 11 |
|  |  | 23.4.16 | 20-50 | 33 | 78 | 13 | 13 |  | 3B |  | 3B |
|  |  | 30.8.16 | 20 | 69 | 80 | 20 | 19 | 6B | 4B |  | 10B |
|  |  | 2016 transect totals | | | | | | 6 | 7 |  | 13 |
|  |  | **Transect totals (range)** | | | | | | **6** | **17** | **1** | **24 (1-10)** |
| *Fig*. 3D  Woodland  (low canopy) | Oak, beech, ash; grass, bluebells, brambles; dense grass | 10.5.15 | nc | 60 | 68 | 17 | 16 |  | 2B |  | 2B |
|  |  | 19.6.15 | 75 | 49 | 50 | 18 | 19 |  | 3B |  | 3B |
|  |  | 2015 transect totals | | | | | |  | 5 |  | 5 |
|  |  | 23.4.16 | 24-87 | 38 | 80 | 11 | 10 | 3B | 2C, 4B |  | 2C, 7B |
|  |  | 30.8.16 | 80 | 76 | 82 | nc | 20 |  |  |  | 0 |
|  |  | 2016 transect totals | | | | | | 3 | 6 |  | 9 |
|  |  | **Transect totals (range)** | | | | | | **3** | **11** |  | **14 (0-9)** |
| *Fig*. 3E  Woodland  (footpath) | Beech, oak; hawthorn, holly; dense grass | 10.5.15 | nc | 61 | 61 | 16 | 16 |  | 2B |  | 2B |
|  |  | 19.6.15 | 30 | 57 | 62 | 19 | 19 |  |  |  | 0 |
|  |  | 2015 transect totals | | | | | |  | 2 |  | 2 |
|  |  | 23.4.16 | 28 | 47 | 82 | 10 | 10 | 1B | 3B | 1♀B | 5B |
|  |  | 30.8.16 | 15 | 74 | 77 | 19 | 19 | 1B | 2B |  | 3B |
|  |  | 2016 transect totals | | | | | | 2 | 5 | 1 | 8 |
|  |  | **Transect totals (range)** | | | | | | **2** | **7** | **1** | **10 (0-5)** |
| *Fig*. 3F  Woodland  (multi-level canopy) | Beech, oak; holly, ferns; beach and oak mast and leaves | 10.5.16 | 0 | 66 | 74 | 16 | 16 |  | 4B* |  | 4B |
|  |  | 19.6.15 | 30 | 55 | 59 | 19 | 19 |  | 2B |  | 2B |
|  |  | 2015 transect totals | | | | | |  | 6 |  | 6 |
|  |  | 23.4.16 | 20 | 41 | 75 | 10 | 9 |  | 8B |  | 8B |
|  |  | 30.8.16 | 10-30 | 74 | 80 | 20 | 20 | 19B | 2B |  | 21B |
|  |  | 2016 transect totals | | | | | | 19 | 10 |  | 29 |
|  |  | **Transect totals (range)** | | | | | | **19** | **16** |  | **35 (2-21)** |
| Transect totals  (range, IQR) | | | | | | | | **35** | **82** | **1♀, 3♂** | **121 (10-35,**  **10-30)** |
| Extras | | 2015 (all B) | | | | | | 29 | 54 | 1♀, 3♂ | 87 |
|  |  | 2016 (all B) | | | | | | 71 | 50 | 1♀ | 122 |
|  |  | **Total extra ticks collected** | | | | | | **100** | **104** | **2♀, 3♂** | **209** |
| Total ticks collected at site | | | | | | | | **135** | **186** | **3♀, 6♂** | **330** |
