# Supplementary material for: Tick hazard in the South Downs National Park (UK): species, distribution, key locations for future interventions, site density, habitats
Source: PeerJ. 2024 Jun 12;12:e17483. doi: 10.7717/peerj.17483 (PMC11179636; doi:10.7717/peerj.17483)
Supplement: Supplemental Information 5 — Ticks were collected through combined sampling with woollen blanket (B), chap (C), and flags (F). *1 removed for safety validation before light microscopy confirmation of ID. NC=not collected. SDW = South Downs Way national trail. [file peerj-12-17483-s005.docx]

| Plot  and  Habitat | Dominant vegetation; under-growth;  main litter | Date | Undergrowth (height cm) | Rh % | | T ^O^C | | Ticks Collected  (*I. ricinus*) | | | |
| --- | --- | --- | --- | --- | --- | --- | --- | --- | --- | --- | --- |
|  |  |  |  | **50cm** | **Litter** | **50cm** | **Litter** | **Larvae** | **Nymphs** | **Adults** | **Totals** |
| *Fig*. 3G  Conifer-Heath | Conifers (recently planted); dense grass; no visible litter | 15. 5.15 | 50 | 55 | 78 | 18 | 23 |  | 2B*,  1C |  | 2B, 1C |
|  |  | 15.6.15 | 75 | 66 | 72 | 15 | nc |  |  |  | 0 |
|  |  | 2015 transect totals | | | | | |  | 3 |  | 3 |
|  |  | 15.11.16 | 100-200 | 56 | 82 | 17 | 16 |  |  |  | 0 |
|  |  | 2016 transect totals | | | | | |  |  |  | 0 |
|  |  | **Transect totals (range)** | | | | | |  | **3** |  | **3 (0-3)** |
| *Fig*. 3H  Conifer-heath border  (track) | Ferns, brambles; dense grass; fern leaves | 15.5.15 | 30 | 60 | 70 | 15 | 15 |  | 7B |  | 7B |
|  |  | 15.6.15 | nc | nc | nc | nc | nc |  | 1B |  | 1B |
|  |  | 2015 transect totals | | | | | |  | 8 |  | 8 |
|  |  | 15.11.16 | 10-120 | 55 | 83 | 16 | 16 |  |  |  | 0 |
|  |  | 2016 transect totals | | | | | |  |  |  | 0 |
|  |  | **Transect totals (range)** | | | | | |  | **8** |  | **8 (0-7)** |
| *Fig*. 3I  Woodland  (very dense canopy) | Conifers; no undergrowth; pine needles and branches | 15.5.15 | 0 | nc | nc | nc | Nc |  | 5B |  | 5B |
|  |  | 15.6.15 | 0 | 80 | 81 | 14 | 14 |  | 4B |  | 4B |
|  |  | 2015 transect totals | | | | | |  | 9 |  | 9 |
|  |  | 15.11.16 | 0-50 | 59 | 82 | 59 | 14 |  | 1B |  | 1B |
|  |  | 2016 transect totals | | | | | |  | 1 |  | 1 |
|  |  | **Transect totals (range)** | | | | | |  | **10** |  | **10 (1-5)** |
| *Fig*. 3J  Downland  (field bordering wood) | Grass (sheep grazed); dense grass; no visible litter | 15.5.15 | 50 | 57 | 73 | 14 | 14 |  |  | 1♂B | 1B |
|  |  | 15.6.15 | nc | 52 | 81 | 19 | 17 |  |  |  | 0 |
|  |  | 2015 transect totals | | | | | |  |  | 1 | 1 |
|  |  | 25.9.16 | 10 | 62 | 85 | 17 | 14 |  |  |  | 0 |
|  |  | 2016 transect totals | | | | | |  |  |  | 0 |
|  |  | **Transect totals (range)** | | | | | |  |  | **1** | **1 (0-1)** |
| *Fig*. 3K  Downland  (field bordering wood) | Grass (sheep grazed); dense grass; no visible litter | 15.5.15 | 30 | 63 | 76 | 15 | 16 |  |  |  | 0 |
|  |  | 15.6.15 | nc | 69 | 80 | 15 | 16 |  |  |  | 0 |
|  |  | 2015 transect totals | | | | | |  |  |  | 0 |
|  |  | 25.9.16 | 10 | 56 | 89 | 17 | 16 |  |  |  | 0 |
|  |  | 2016 transect totals | | | | | |  |  |  | 0 |
|  |  | **Transect totals (range)** | | | | | |  |  |  | **0 (0-0)** |
| *Fig*. 3L  Downland  (SDW footpath) | Nettles; dense grass; no visible litter | 15.5.15 | nc | 78 | 73 | 14 | 12 |  |  |  | 0 |
|  |  | 15.6.15 | 120 | 66 | 76 | 18 | 20 |  |  |  | 0 |
|  |  | 2015 transect totals | | | | | |  |  |  | 0 |
|  |  | 25.9.16 | 100 | 59 | 90 | 16 | 16 |  | 1B |  | 1B |
|  |  | 2016 transect totals | | | | | |  | 1 |  | 1 |
|  |  | **Transect totals (range)** | | | | | |  | **1** |  | **1 (0-1)** |
| Site transect totals  (mean, range, IQR) [Three samplings only, due to a 2016 access restriction] | | | | | | | |  | **22** | **1♂** | **23 (4,**  **0-10, 1-9)** |
| Extras | | 2015 (all B) | | | | | |  | 2 |  | 2 |
|  |  | 2016 (all B) | | | | | | 3 | 3 |  | 6 |
|  |  | **Total extra ticks collected** | | | | | | **3** | **5** |  | **8** |
| Total ticks collected at site | | | | | | | | **3** | **27** | **1♂** | **31** |
