# Supplementary figures and images for: Tick hazard in the South Downs National Park (UK): species, distribution, key locations for future interventions, site density, habitats
Source: PeerJ. 2024 Jun 12;12:e17483. doi: 10.7717/peerj.17483 (PMC11179636; doi:10.7717/peerj.17483)

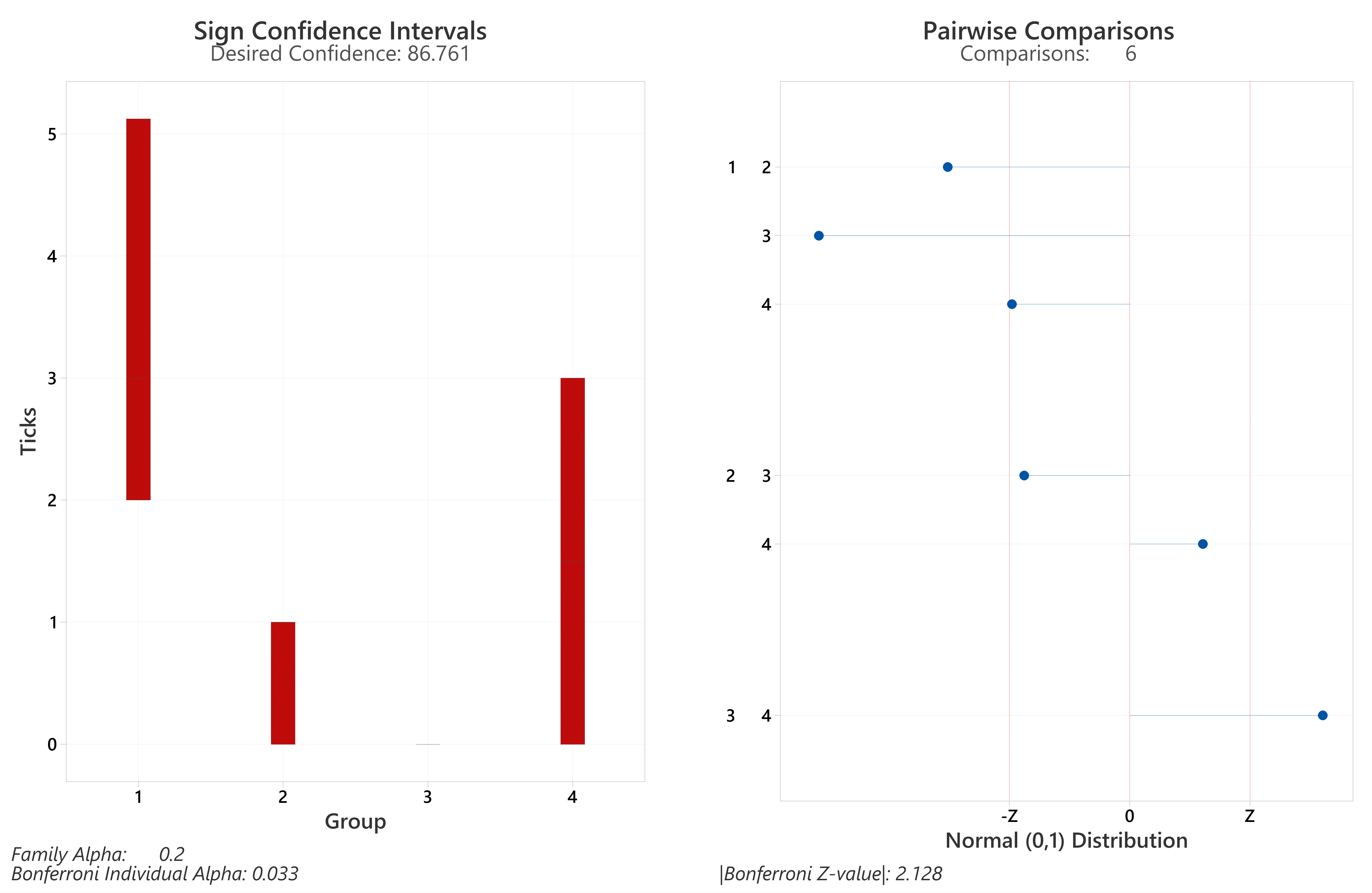

Supplement: Supplemental Information 6 — N=90. 1=The Mens; 2=Cowdray Estate; 3=Seven Sisters Country Park; 4=Queen Elizabeth Country Park. [file peerj-12-17483-s006.png]

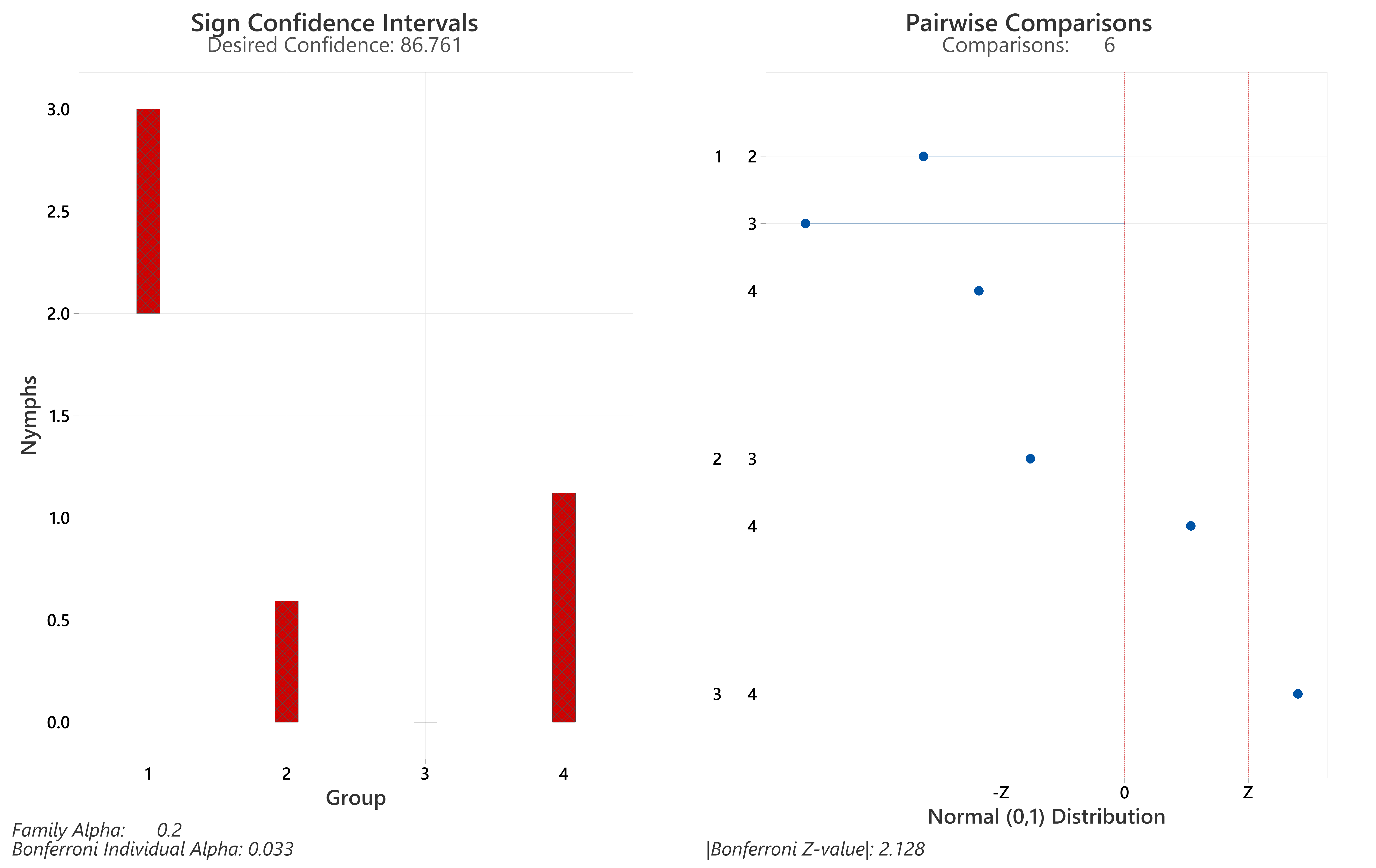

Supplement: Supplemental Information 7 — 1=The Mens; 2=Cowdray Estate; 3=Seven Sisters Country Park; 4=Queen Elizabeth Country Park. [file peerj-12-17483-s007.png]

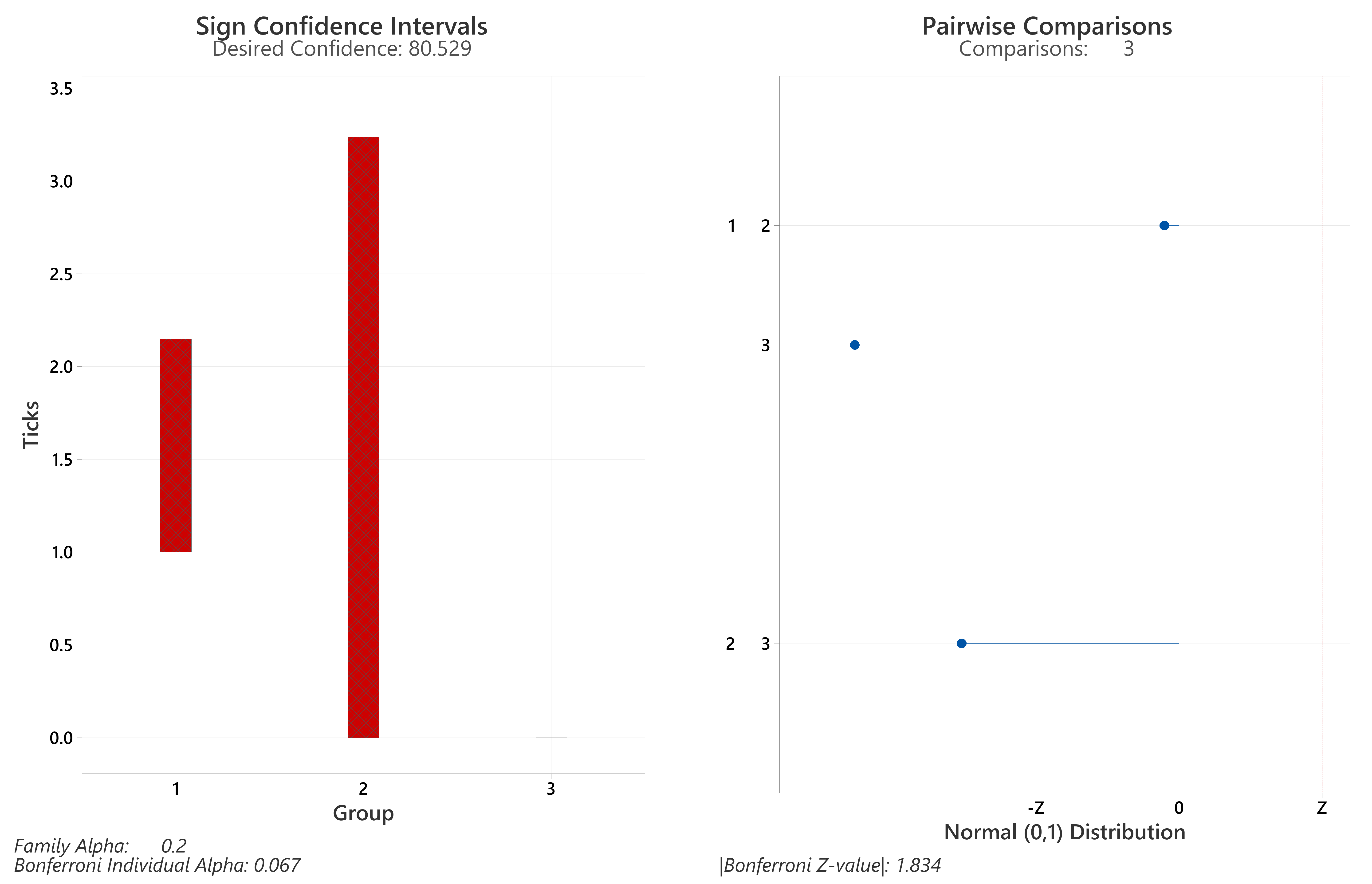

Supplement: Supplemental Information 8 — N=90. 1=deciduous woodland; 2=Conifer woodland/planting; 3=Sheep grazed downland. [file peerj-12-17483-s008.png]

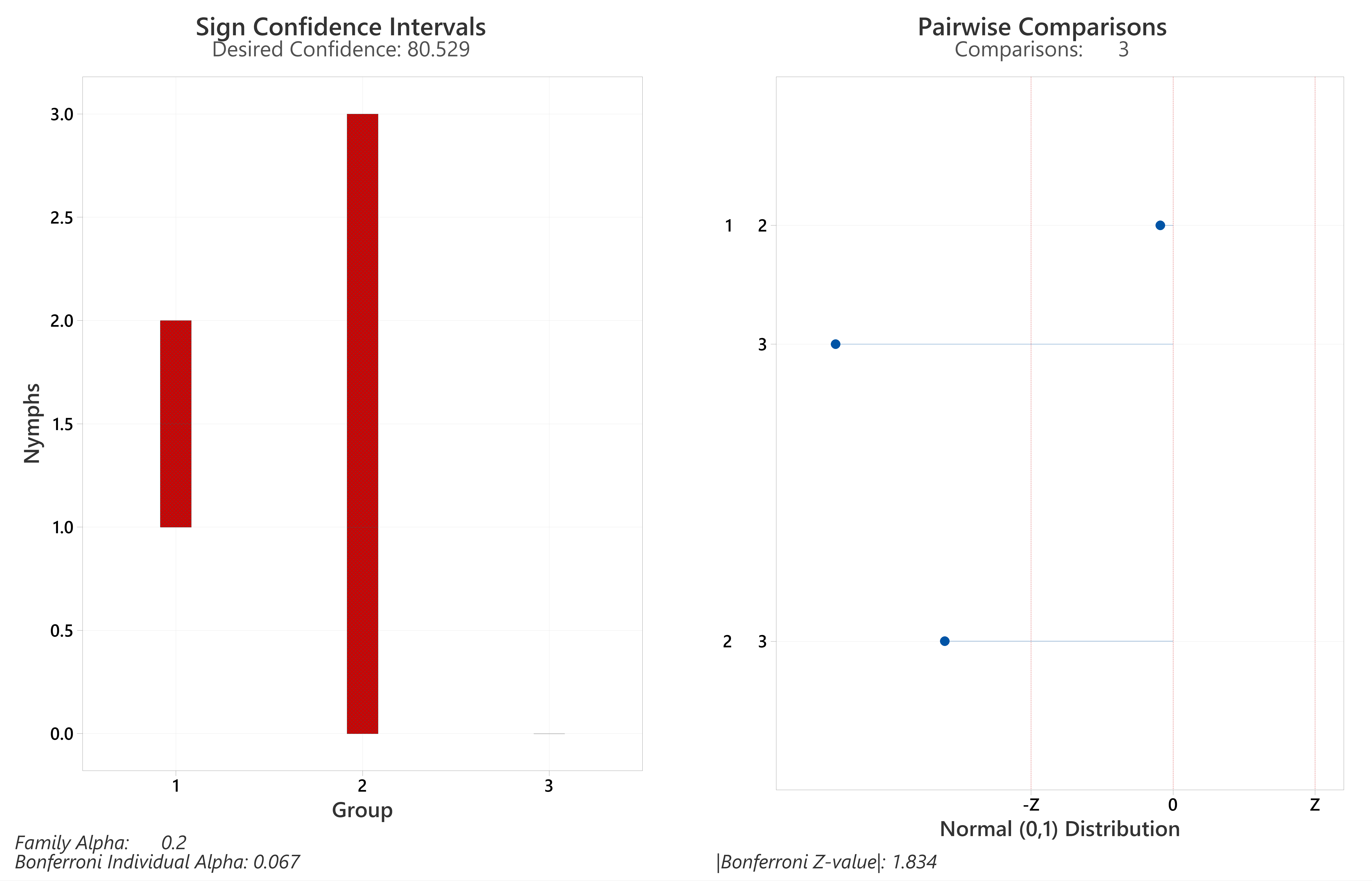

Supplement: Supplemental Information 9 — N=90. 1=deciduous woodland; 2=Conifer woodland/planting; 3=Sheep grazed downland. [file peerj-12-17483-s009.png]
